# Mechanochromic, Low‐Cost, and Structurally Colored Displays Using Biodegradable Hydroxypropyl Cellulose

**DOI:** 10.1002/adma.202418880

**Published:** 2025-05-09

**Authors:** Charles H. Barty‐King, Maxime Burgonse, Silvia Vignolini, Jeremy Baumberg, Michael De Volder

**Affiliations:** ^1^ Institute for Manufacturing Department of Engineering University of Cambridge 17 Charles Babbage Road Cambridge CB3 0FS UK; ^2^ Yusuf Hamied Department of Chemistry University of Cambridge Lensfield Road Cambridge CB2 1EW UK; ^3^ NanoPhotonics Centre Department of Physics Cavendish Laboratory University of Cambridge Cambridge CB3 0US UK

**Keywords:** cholesteric liquid crystals, hydroxypropyl cellulose, mechanochromism, pixels, reflective display

## Abstract

Mechanochromic materials have garnered significant interest over the past decade due to their ability to change color in response to mechanical cues. While it is known that hydroxypropyl cellulose (HPC) self‐assembles into biodegradable and low‐cost mechanochromic materials, with a wide range of applications from edible colorants to optical strain sensors, mechanochromic HPC displays themselves are not reported. Here we address this challenge by combining thin mechanochromic HPC films with microfluidic arrays of inflatable microactuators that exert controlled forces. With these devices, the mechanochromic strain sensitivity, color resolution, response times, and operating frequencies of photonic aqueous HPC films are measured at decreasing length scales for the first time. Various pixel sizes, geometry, and input frequencies are also assessed to investigate mechanochromic HPC as a potential low‐cost, biodegradable display. Potential applications range from dynamic color pixels for soft robotics to more environmentally responsible RGB display technology.

## Graphical abstract

Mechanochromic hydroxypropyl cellulose (HPC) integrated with microfluidic devices creates scalable, eco‐friendly reflective color displays. We demonstrate mechanochromic displays with 500 µm pixel size and 5Hz switching rates with room for optimisation. The proposed mechanochromic HPC displays are an initial step toward more environmentally responsible color display and pixel technologies.

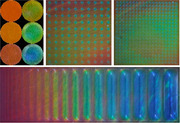

## 1 Introduction

Hydroxypropyl cellulose (HPC) is well established in the medical, pharmaceutical, and food industries as a widely applicable non‐toxic, and cost‐effective raw material.^[^ ^1^, ^2^, ^3^, ^4^, ^5^, ^6^, ^7^, ^8^, ^9^, ^10^, ^11^, ^12^, ^13^, ^14^, ^15^ ^]^ More recently however, HPC has gained interest as an applied photonic material specifically,^[^ ^16^, ^17^, ^18^, ^19^, ^20^, ^21^ ^]^ due to its remarkable mechanochromic properties (i.e., changes of color with strain).^[^ ^22^, ^23^, ^24^, ^25^ ^]^ Shown to have great potential for optical strain sensing applications,^[^ ^16^, ^17^, ^18^, ^26^, ^27^, ^28^ ^]^ mechanochromic HPC can be manufactured using continuous roll‐to‐roll (R2R) production,^[^ ^21^ ^]^ and retains favorable shear‐thinning rheology in combination with other bio‐polymers.^[^ ^20^, ^29^, ^30^, ^31^ ^]^ Furthermore while other examples of non‐toxic mechanochromic liquid crystals exist,^[^ ^18^, ^32^, ^33^, ^34^, ^35^, ^36^, ^37^, ^38^, ^39^, ^40^, ^41^, ^42^, ^43^ ^]^ the production of photonic HPC is comparatively simple in producing intrinsically mechanochromic materials that are simultaneously biodegradable, edible and scalable.^[^ ^16^, ^20^ ^]^ A rich seam of exploration has therefore begun in the application of mechanochromic HPC devices.^[^ ^16^, ^17^, ^18^, ^20^, ^21^, ^26^, ^27^, ^44^, ^45^, ^46^, ^47^ ^]^ Typically HPCs cholesteric pitch, which derives its mechanochromic structural coloration, is placed under a compressive strain and the resulting color change used as an optical strain sensor – in combination with smartphones, cameras or microscopes.^[^ ^16^, ^17^, ^18^, ^20^, ^21^, ^26^, ^44^ ^]^ Interestingly however, there is no literature on the “inverse” device, where fine control of the strain is used to create an HPC color display.^[^ ^48^, ^49^, ^50^, ^51^, ^52^, ^53^ ^]^ We address this challenge through the development of microactuator arrays using soft lithography and microfluidics.^[^ ^54^, ^55^, ^56^ ^]^

Polydimethylsiloxane (PDMS) allows for optically transparent and biocompatible replica‐mould microstructures with high mechanical flexibility and fidelity (i.e., soft‐lithography).^[^ ^54^, ^55^, ^57^, ^58^, ^59^, ^60^, ^61^, ^62^, ^63^ ^]^ Here we utilize PDMS membranes to manufacture balloon‐type microactuators that can be pneumatically inflated through embedded microchannels (i.e., microfluidics).^[^ ^54^, ^55^, ^57^, ^58^ ^]^ The magnitude of displacement is controlled via membrane geometry, thickness, and driving pressure.^[^ ^53^, ^55^ ^]^ These devices enable fine control of the mechanical strain applied to thin HPC films and a proof of concept for the fabrication of mechanochromic HPC displays using microactuating forces. Using individual and oscillating pressure steps, we report the dynamic mechanochromic strain sensitivity, response times, and frequency responses of mechanochromic HPC in a pixel‐display context for the first time. We also investigate the initial specifications that mechanochromic HPC‐based displays achieve and explore the emergent key principles for their further development and optimization. Lastly, we report a strain threshold at which a viscoelastic HPC pitch compression regime gives way to a more flowing regime at ≈25–27% applied strain.

**Figure 1:**
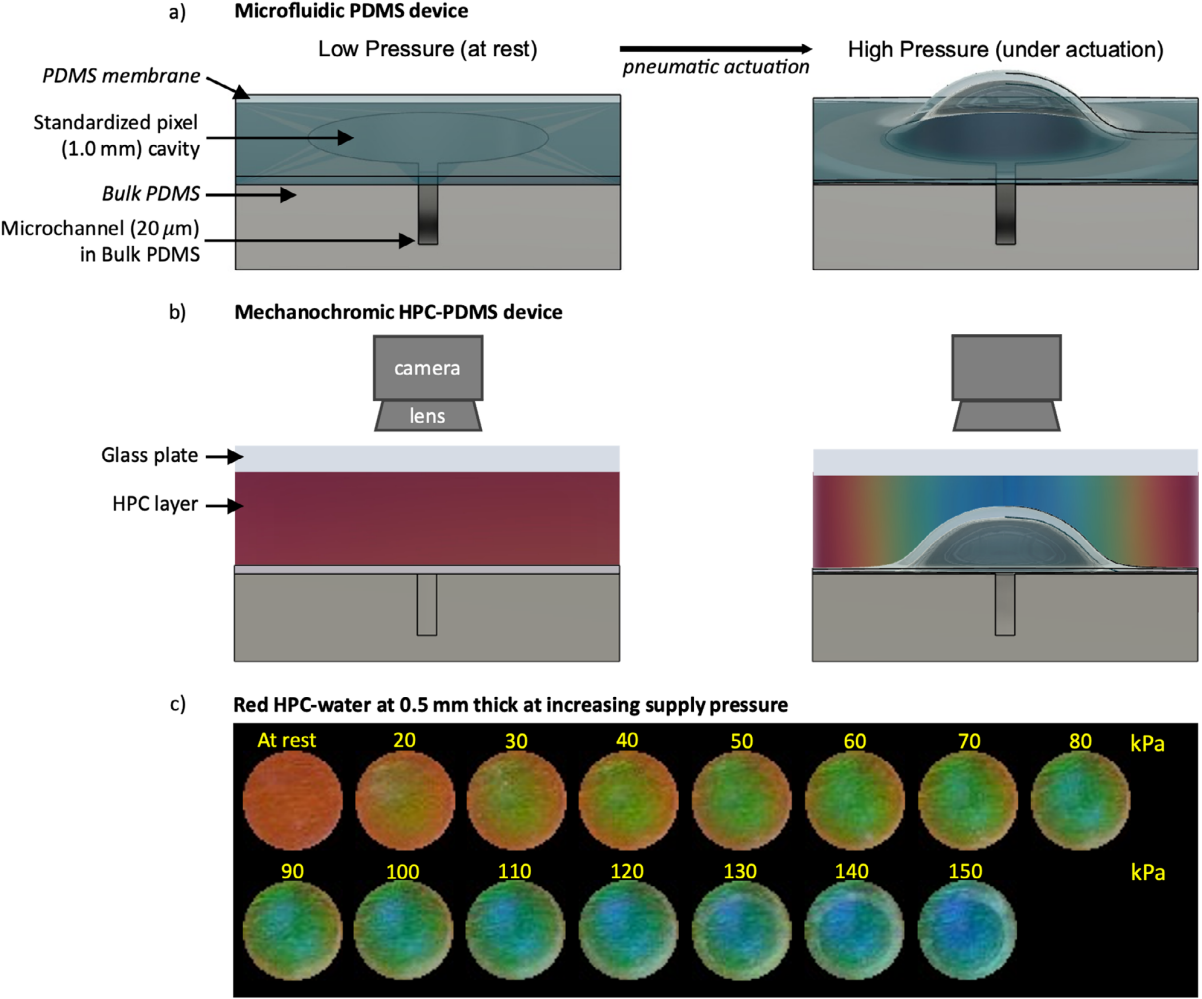
a) Cross‐sectional 3D schematic representation of the microfluidic PDMS device. Not to scale. b) Cross‐sectional 2D schematic working principle of the completed mechanochromic HPC PDMS device after a continuous HPC film has been sealed against the PDMS with a glass plate. Not to scale. (a,b) At rest (left) and under pneumatic actuation (right). The PDMS membrane actuates HPC against the glass plate and is observed by the camera. c) Pictures of the mechanochromic response of 0.5 mm thick HPC within the region of interest (the standardized pixel) under increasing supply pressure, exemplifying the maximum change in color (max ΔHue) at each applied pressure. Circles are 1 mm in diameter.

Microactuators are fabricated by spin‐coating thin PDMS membranes and bonding them to lithographic‐patterned PDMS substrates, termed “bulk PDMS,” with pre‐designed cavities and microchannels (**Figure** **1**a). Continuous HPC films with thicknesses of 0.5, 1.0, and 1.5 mm are subsequently coated on top of the PDMS membrane of three independent devices, using a spacer to control film thickness, and sealed with a glass plate. The microactuators then compress the HPC locally against the glass plate (Figure 1b), with the resultant mechanochromism recorded optically with a color USB camera (Figure 1c). With the camera positioned normal to the sample surface, color values are reported as Hue (degrees°) by utilizing the RGB (red, green, blue) to HSL (hue, saturation, lightness) color space transformation.^[^ ^21^, ^64^, ^65^ ^]^ The HPC coloration at rest is chosen to be red (35 wt.% H_2_O) for all experiments, unless otherwise stated, so that a compressive blueshift is observed through the full visible spectrum. The same principle applies to the UV and IR wavelength ranges and tensive forces.

Full experimental details, definitions, HPC formulation procedure, setup and control systems (Figure S1, Supporting Information), as well as the manufacture of the mechanochromic HPC‐PDMS device (Figures S2–S5, Supporting Information), are provided in the Experimental section below and Supporting Information, respectively. An investigation of the achievable color ranges of HPC under compressive pressure events is provided by Liang et al.^[^ ^21^ ^]^ A good linearity also exists between hue values (°) and spectral wavelengths (nm).^[^ ^64^ ^]^

## 2 Results and Discussion

### 2.1 Mechanochromic Strain Sensitivity

**Figure 2:**
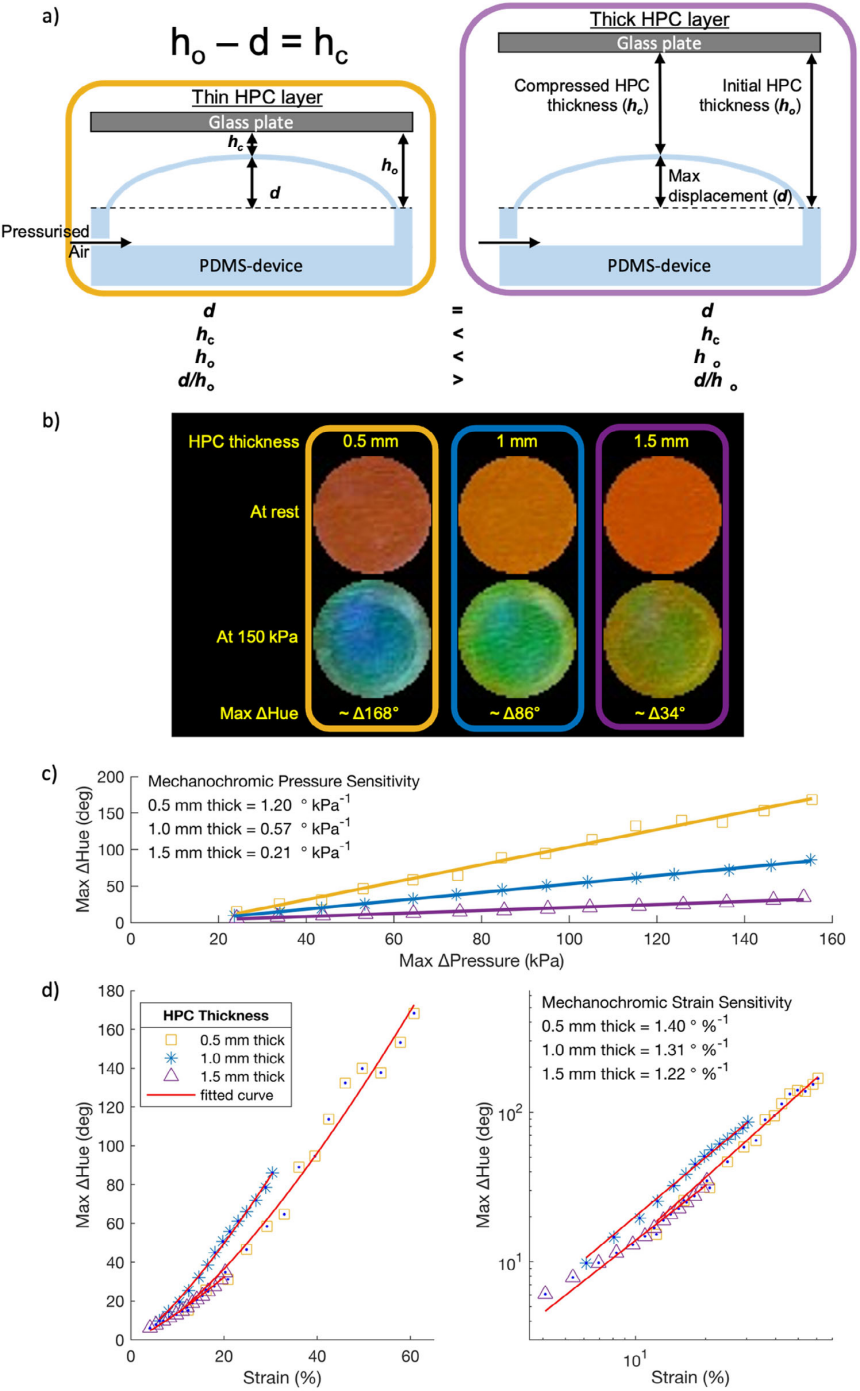
Mechanochromic HPC‐PDMS devices at varying HPC thicknesses: yellow line and squares are 0.5 mm thick HPC, blue line and asterisk are 1.0 mm thick HPC, purple line and triangles are 1.5 mm thick HPC. a) Schematic representation of the comparative PDMS displacements of two different HPC thicknesses. b) Pictures of the standardized pixel at rest and at the max ΔHue (150 kPa) for each HPC thickness. Circles are 1 mm. c) Max ΔHue (°) versus max measured supply pressure (kPa) displaying the slope coefficient of each plot, and d) max ΔHue (°) versus strain (%) for all HPC thicknesses with linear axis (left) and logarithmic axes displaying the slope coefficients of each plot (right).

The color‐pressure response of a 0.5 mm thick HPC film is shown in Figure 1c. As the supply pressure is increased in 10 kPa increments up to 150 kPa, a circular 1.0 mm diameter microactuator is inflated to compress the HPC cholesteric pitch and induce a reversible mechanochromic blueshift, termed the “standardized pixel.”^[^ ^16^, ^23^, ^66^, ^67^ ^]^ A non‐uniform structural color is observed across the HPC pixel (Figure 1c) and attributed to the constrained arc‐like displacement of the PDMS membrane (Figures 1b, and **2**a).^[^ ^68^ ^]^ While the displacement of the PDMS membrane increases as a function of the applied supply pressure (Figure S6, Supporting Information, measured in air), comparative PDMS displacements (*d*) cause the applied strain (*ε*) within a device to increase as HPC thickness decreases (*ε  =  *| *‐d / h_o _* |), following:

(1) $$\varepsilon =\frac{h_{c}-h_{o}}{h_{o}}=\left|\frac{-d}{h_{o}}\right|$$

where *h_c_* = minimum thickness of HPC during compression, *h_o_* = initial HPC thickness at rest, and *d* = microactuator displacement (Equation (1)).^[^ ^69^ ^]^ Therefore, for a given displacement *d*, thinner HPC layers experience a greater applied strain and therefore a larger color shift (Figure 2a). This is validated in Figure 2b where a maximum supply pressure of 150 kPa induces an HPC color change (ΔHue in °) of ≈Δ168° for 0.5 mm thick HPC (yellow line), ≈Δ86° for 1.0 mm thick (blue line) and ≈Δ34° for 1.5 mm thick HPC (purple line).

Taking the linear slope of the maximum ΔHue versus applied supply pressure for each HPC thickness over the full pressure range, the mechanochromic *pressure* sensitivity in our devices is provided (Figure 2c): 1.20 ° kPa^−1^ for 0.5 mm thick, 0.57 ° kPa^−1^ for 1.0 mm thick, and 0.21 ° kPa^−1^ for 1.5 mm thick HPC. When undergoing similar displacements, the magnitude of HPCs mechanochromism increases with decreasing HPC thickness.

The relationship between the strain and resultant color change is extracted using the microactuator displacements of Figure S6 (Supporting Information), used to determine HPCs mechanochromic *strain* sensitivity (Figure 2d). For all our devices the relationship seems to follow similar trends that can be fitted to a power curve relationship (Figure 2d; Figure S6 and Equation S1, Supporting Information), providing the mechanochromic strain sensitivity at different HPC thicknesses: ≈1.40 ± 0.18 ° %^−1^ at 0.5 mm thick, ≈1.31 ± 0.06 ° %^−1^ at 1.0 mm thick, and ≈1.22  ± 0.10 ° %^−1^ at 1.5 mm thick HPC, respectively (± ranges determined from 95% confidence bounds). With overlapping confidence bounds from three independent devices, we report the relationship (averaged) between strain and HPC mechanochromism to follow (Equation (2)):

(2) $$\Delta Hue=a\times \varepsilon^{1.31\pm 0.11}$$

where *a* = ΔHue at 1% strain and *ε* = high strain > ≈25%. The mechanochromic sensitivity of aqueous HPC is therefore found to be non‐linear to applied strain and independent of HPC thickness at the regimes measured (<1.5 mm thick HPC). Color histograms and chromaticity diagrams can also be found in Figure S7 (Supporting Information) to demonstrate the color ranges we achieved.

Note that for the practicalities of analysis the applied strain presented here is an approximation that assumes: 1) the magnitude of the PDMS membrane displacement is not influenced by the presence of HPC, 2) HPC experiences no lateral flow during actuation, and 3) the maximum displacement (*d*) occurs across the whole pixels area. These assumptions limit the true accuracy of our results, representing only a valuable first initial insight into the relationship between color and strain and likely practical limitations of mechanochromic HPC displays. Further investigation is required to understand the exact relationship between strain, color change, and rheology. A discussion of the strain and associated assumptions are provided in more detail in Figure S6 (Supporting Information). In summary, as photonic HPC is a liquid system, some degree of inelastic lateral flow during actuation is inevitable.^[^ ^16^, ^20^, ^21^ ^]^ Combined with its shear‐thinning rheology it follows that the magnitude of inelastic lateral flow will increase with strain,^[^ ^20^, ^21^, ^30^ ^]^ meaning some relaxation of the color is to be expected during high strain actuations. This behavior is observed in Figure 2d where the linear axes (left) have a linear behaviour at high strains and curved behavior at low; alternatively with logarithmic axes (right), showing a good fit at high strain but poor fit at low. It is therefore impossible to capture the entire behavior with one equation. Our analysis as discussed when reporting our device response times below, shows that this HPC reflow appears when strains exceed ≈25–27%. The addition of a gelling agent such as gelatin does not negatively impact HPCs ability to display mechanochromism or shear‐thinning (as well as broadening its dynamic color range),^[^ ^20^ ^]^ and could therefore be used to optimize HPCs flow properties and strain sensitivity for a given application or actuator type, discussed later. Finally, the rheology of HPC is highly complex for determining its liquid crystalline behavior, application and optical effect, and has been studied extensively.^[^ ^16^, ^20^, ^21^, ^24^, ^29^, ^30^, ^31^, ^47^, ^63^, ^67^, ^70^, ^71^, ^72^ ^]^ While outside the scope of this paper, a deep rheological analysis of mechanochromic HPC in a strain‐display context would represent significant value to the body of work.

### 2.2 Pixel Size and Shape

**Figure 3:**
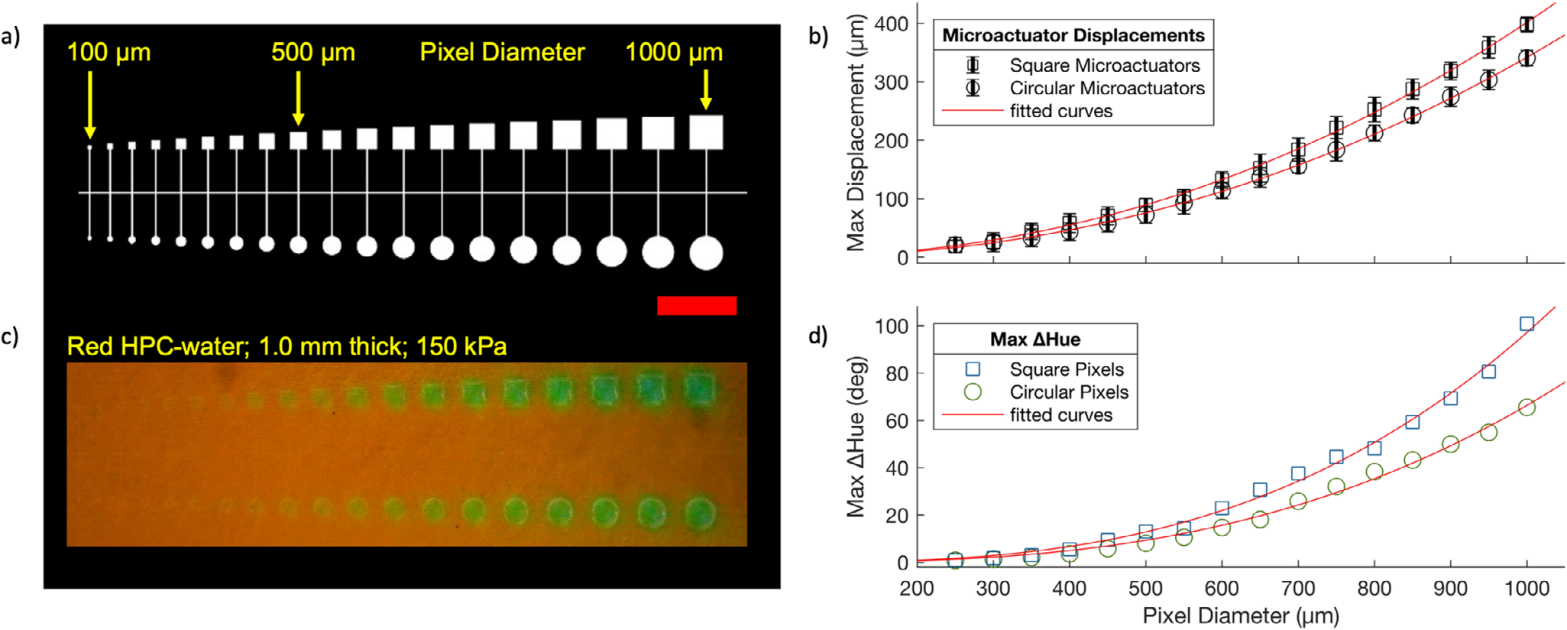
One cohesive architecture with two pixel geometries (circles and squares) descending in diameter right‐to‐left from 1000 to 100 µm in 50 µm increments. Red scale bar is 2.5 mm. a) Checkplot (white) is to scale and is the microfluidic design. b) Microactuator displacement (µm) versus pixel size (µm). Error bars indicate the standard deviation in microactuator displacement in air. c,d) Red HPC‐water (35 wt.% H2O) at 1.0 mm thick during a 150 kPa actuation. c) Picture at max ΔHue, and d) a plot of max ΔHue (°) versus pixel size (µm).

We explore the effect of pixel size and shape (circular and square) on HPCs mechanochromism when applying a 150 kPa supply pressure (**Figure** **3**). Pixel diameters increasing from 100 to 1000 µm in 50 µm increments are used to drive a 1 mm thick HPC film.

An increase in pixel diameter results in a larger displacement of the actuator membrane for the same applied pressure, as expected from shell theory,^[^ ^73^, ^74^ ^]^ and shown in Figure 3a,b for both round and square pixels. In turn, the increased displacement causes an increase in the max ΔHue (Figure 3c,d). HPCs color change can therefore be controlled by both pixel size and shape from one pneumatic air supply, in addition to the supply pressure and HPC thickness (i.e., strain).

The smallest pixels showing a color change are ≈400–500 µm in diameter. Pixels smaller than ≈300 µm failed to generate sufficient PDMS displacement and therefore strain in the HPC, resulting in no observable color change with the camera used in this paper. While this could be overcome with thinner HPC films or higher driving pressures (i.e., increasing the strain), the latter often led to failure of the bond between the PDMS membrane and bulk PDMS substrate in our devices. A key challenge in achieving HPC mechanochromism while shrinking device volume (i.e., reducing pixel diameter and HPC thickness simultaneously) is therefore likely to be the consistent and reliable application of strain, by actuators, onto HPC as length scales decrease.

### 2.3 Response Times

**Figure 4:**
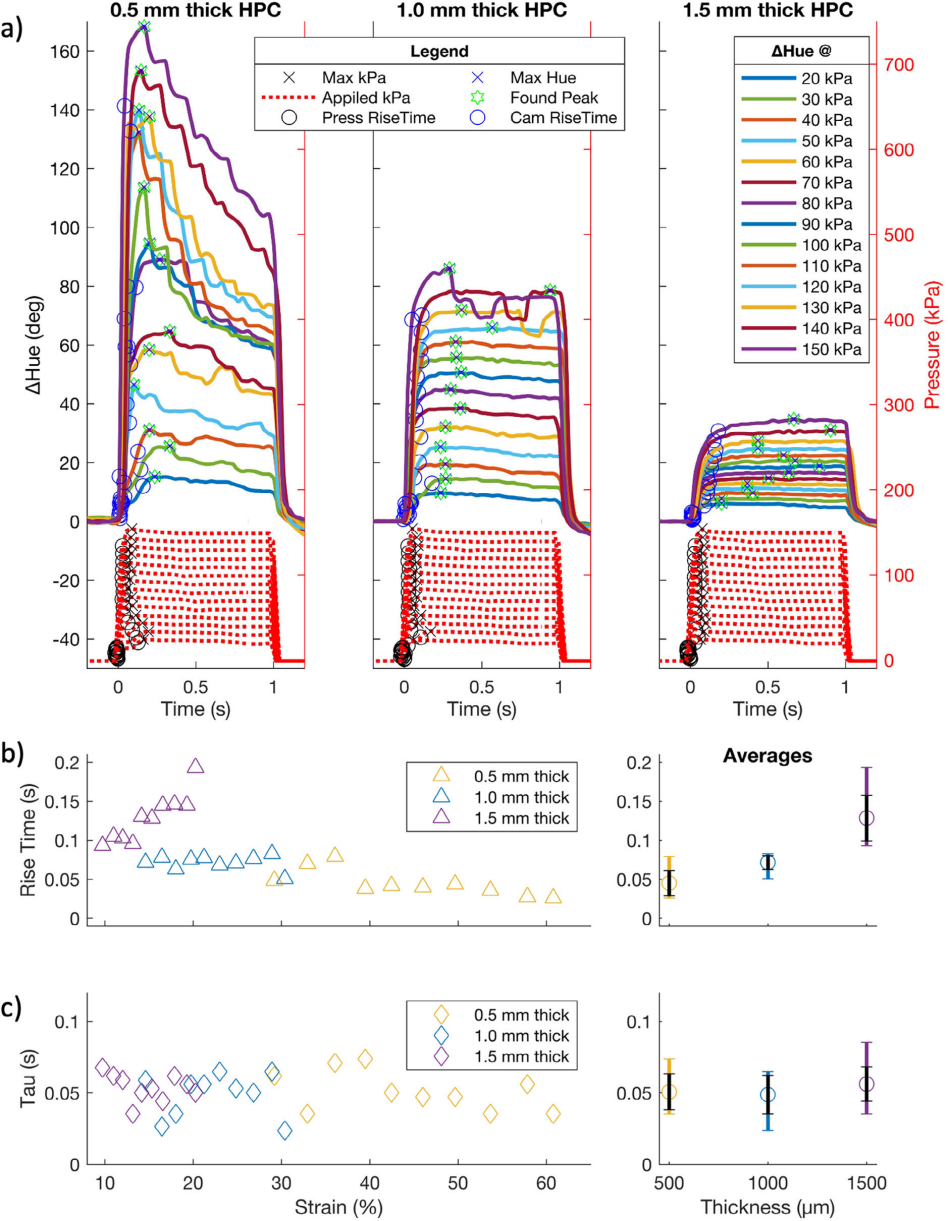
Time responses of HPC mechanochromism (solid lines) at three thicknesses during independent single square pulses of 1 s (dotted red lines) between 20 and 150 kPa in 10 kPa intervals. a) ΔHue (°) versus time (s) for all experiments. Circles: rise time variables at 10% and 90% step height of each plot. Blue crosses: max Hue achieved. Green Stars: fitted peak. Black Crosses: max kPa reached. b,c) Response times achieved in (a) as a function of strain (%) with HPC thickness represented in yellow (0.5 mm), blue (1.0 mm) and purple (1.5 mm), with averages shown on the right‐hand side. Colored error bars are max/min range. Black error bars are the standard deviation. (b) Rise time (s). (c) Mechanochromic relaxation time constant (s).

The response times of our devices are investigated using the standardized pixel introduced above. Using a range of pressure steps different strains are applied to HPC for 1 s, and the device relaxed (**Figure** **4**). The ΔHue at each supply pressure is measured over time (Figure 4a), and the rise times and mechanochromic relaxation time constant (*τ*) for each HPC thickness plotted against strain (Figure 4b,c, respectively). All response times resulting from a 50 kPa supply pressure and below are omitted from Figure 4b,c due to the control valve struggling to provide a consistently rapid response at low pressure (Figure 4a; dotted red line).

The ΔHue relaxes for the 0.5 mm thick HPC above 50 kPa, and the 1 mm thick HPC above 130 kPa, despite the pressure step still being actuated (Figure 4a; Figure S8, Supporting Information). For the 0.5 mm thick HPC device, a pressure of ≈50 kPa corresponds to a displacement of ≈124 µm (Figure S6, Supporting Information), resulting in a strain of ≈25%. Similarly, for the 1.0 mm thick HPC device, a pressure of ≈130 kPa corresponds to a displacement of ≈268 µm and a strain of ≈27%. This suggests a strain threshold ≈25–27%. Above this threshold, HPC appears to more readily flow under the applied strain.^[^ ^20^, ^21^, ^30^ ^]^ This could be due to its shear‐thinning rheology, where an increase in strain leads to greater inelastic lateral flow,^[^ ^75^ ^]^ and thus a reduction in ΔHue when under critical shear stress. Regardless, at high strains the pixel is highly sensitive but unstable, while at lower strains sensitivity is reduced but stability increases. This would imply a predominantly viscoelastic response (indicated by the square‐like output signal) giving way to a more flowing, sheared‐like response, indicated by the more instantaneous recovery even during actuation (Figure 4a). The strain threshold may therefore mark a transition in HPCs pitch compression regime, offering potential insights into its complex rheological behavior. Future work could involve optimizing HPCs rheology to withstand higher strains within a display screen context, but is beyond the scope presented here.

Measuring the ΔHue rise time from Figure 4a, averages of ≈45 ± 16 ms for 0.5 mm thick, ≈71 ± 9 ms for 1.0 mm thick, and ≈129 ± 29 ms for 1.5 mm thick HPC devices are observed, with ± as standard deviation (Figure 4b). In our devices, all ΔHue rise times are observed below ≈200 ms. However, these rise times are a combination of our pneumatic control system and the microactuators, as well as the HPC, and therefore do not represent the upper limit of what HPC might achieve. The only other reported rise time of mechanochromic HPC found in the literature states 631 ms when measuring its electromechanical response,^[^ ^44^ ^]^ suggesting our pneumatic microactuators have a higher bandwidth.

The mechanochromic relaxation time constant, *τ*, when the applied strain is removed (Figure S9, Supporting Information) is 52 ± 13 ms averaged across all devices, with ± as standard deviation (Figure 4c). HPCs mechanochromic relaxation time is therefore independent of its thickness and the applied strain within the measured regimes (≤1.5 mm thick HPC up to ≈60% strain).

Ultimately, both the rise time and the mechanochromic relaxation time are functions of the properties of the HPC and mechanical actuators used. Any potential mechanochromic HPC displays are therefore likely to have faster response times with the careful choice of the mechanical actuators, the chosen HPC formulation and its rheology, and the optimization of the device design itself.

### 2.4 Frequency Responses

**Figure 5:**
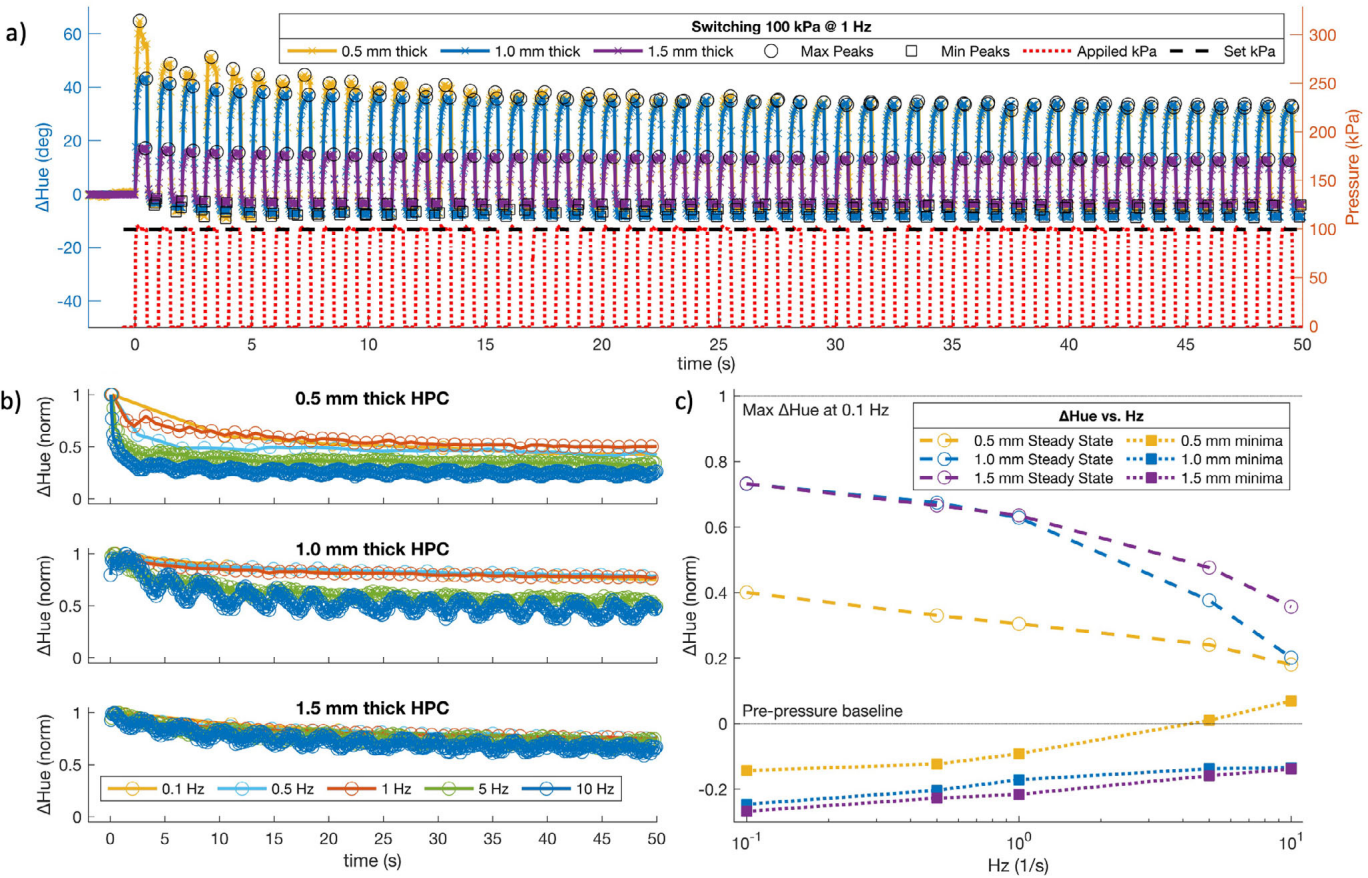
ΔHue (°) at three HPC thicknesses in response to an oscillating 100 kPa square pulse (red dotted line) at 0.1, 0.5, 1, 5, and 10 Hz. Circles and squares represent the maxima and minima peaks of the oscillating ΔHue response, respectively. a) ΔHue through time at 1 Hz. b) Max ΔHue peaks (circles) through time for all experiments. Normalised to the largest maximum ΔHue achieved for each experiment. c) ΔHue versus Hz showing the steady state (circles; dashed line) and minima (squares; dotted line) ΔHue for all experiments. Normalised to the maximum ΔHue achieved at 0.1 Hz. a,c) HPC thicknesses are represented with yellow (0.5 mm), blue (1.0 mm), and purple (1.5 mm) lines and markers.

The frequency responses of HPC in our devices are investigated using cycling conditions and the standardized pixel (**Figure** **5**). A supply pressure of 100 kPa is switched on and off at frequencies of 0.1, 0.5, 1, 5, and 10 Hz, and the amplitude response of ΔHue through time for each HPC thickness recorded (Figure S10, Supporting Information). Maxima (*circles*) and minima (*squares*) ΔHue are denoted for each oscillation, exemplified in Figure 5a. First, the amplitude of ΔHue is normalized to the maximum ΔHue achieved for each experiment (Figure 5b), before being normalized to the maximum ΔHue achieved at the lowest frequency tested (0.1 Hz) for each HPC thickness (Figure 5c).

HPCs mechanochromism is attenuated by a minimum of ≈27–30% max ΔHue under all cycling conditions tested. This is likely due to its shear‐thinning rheology, where the loss factor – the ratio between the loss modulus and storage modulus – increases as angular frequency is increased, reducing viscosity.^[^ ^20^, ^21^, ^29^, ^30^, ^71^ ^]^ HPC struggles to track the input signal, and its mechanochromism becomes attenuated during cycling. The max ΔHue in the 0.5 mm thick HPC device also attenuates during the first ≈25–30 s of operation to a greater degree than the other devices (Figure 5b). Using a supply pressure of 100 kPa, the 0.5 mm thick HPC undergoes a strain of ≈42% (microactuator displacement ≈212 µm; Figure S6, Supporting Information). This resides well above the reported ≈25–27% strain threshold (Figure 2). At such high strains, we suggest that shear‐thinning causes sufficient lateral flow in HPC that its viscosity reduces and becomes attenuated before stabilizing (Figure 5a,b). In contrast, the ≈14% applied strain of the 1.5 mm thick HPC resides well below the strain threshold, with HPCs viscoelasticity preventing immediate reflow, and attenuation over time is largely independent of the frequency applied (Figure 5b; Figure S11b, Supporting Information). Through time, we observe maximum attenuations of ≈30%, ≈50%, and ≈65% for the 1.5, 1.0, and 0.5 mm thick HPC devices, respectively (Figure S11b, Supporting Information).

Within each device, we also observe that the attenuation increases as a function of frequency (Figure 5c), suggesting the color change is becoming limited by the device's bandwidth. When approaching 10 Hz, we observe maximum attenuations of ≈65%, ≈80%, and ≈82% for the 1.5, 1.0, and 0.5 mm thick HPC devices, respectively (Figure 5c). It is clear the interplay between the applied strain and applied frequency is an important consideration for the conceptualization of mechanochromic HPC displays and their application.

It is worth us noting that Figure 5c also shows that under cycling conditions, as frequency increases (with HPC mechanochromism struggling to track the input signal and further limited by the PDMS devices bandwidth), the maxima and minima color change begin to converge toward a common ΔHue value. This is most pronounced in the 0.5 mm thick HPC at 10 Hz. This may indicate a solid‐state pixel coloration is achievable at high enough frequencies, as discussed in Figure S11 (Supporting Information).

### 2.5 Pixel Gradients, Spacing, and Arrays

**Figure 6:**
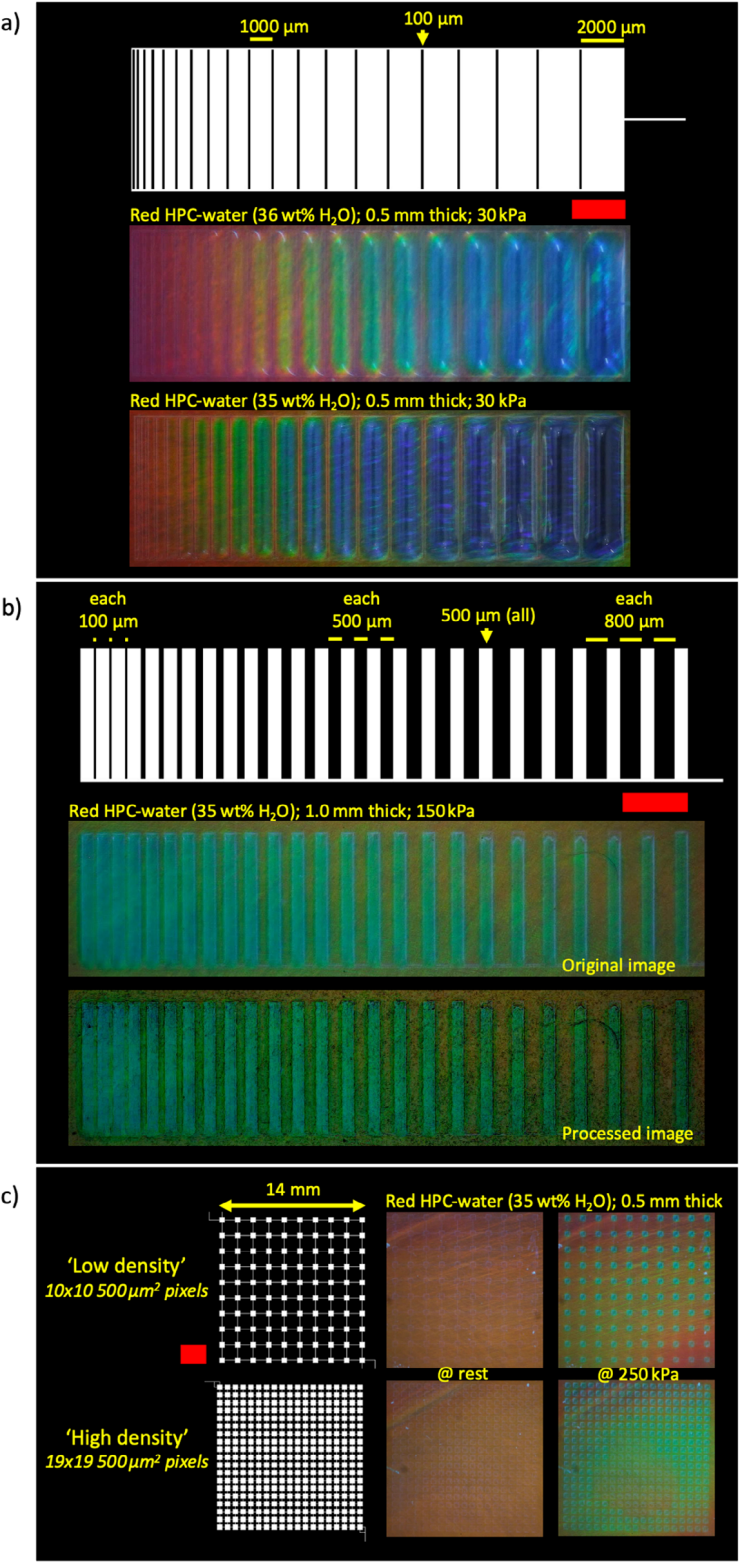
The mechanochromic HPC‐PDMS device in different design configurations. Checkplot (white) is to scale and is the microfluidic design. Red scale bars are 2.5 mm. a) Pixels descend in width from 2000 to 100 µm in 100 µm increments with fixed spacing (100 µm). b) Pixels of fixed width (500 µm) descend in spacing from 800 to 100 µm in 100 µm increments every three pixels right‐to‐left. Top: original RGB image. Bottom: processed RGB image (original – edge filter). c) 14 × 14 mm array of square‐shaped 500 µm^2^ pixels. Left: checkplot. Middle: at rest. Right: at 250 kPa. Top: “low density” 10 × 10 array (1 mm pixel spacing). Bottom: “high density” 19 × 19 array (250 µm pixel spacing).

The interplay between the pixel size, spacing, HPC thickness, and supply pressure for specific effects and detail is investigated (**Figure** **6**). As already shown, larger pixels achieve a greater displacement for the same applied pressure (Figure S6, Supporting Information) and thus a greater strain and color change (Figures 2, and 3).

First, we use this approach for a mechanochromic rainbow effect (Figure 6a). A connected array of pixels with increasing widths and spaced by fixed 100 µm thick PDMS walls produces a change in hue across the display. Conversely, by fixing the pixel width at 500 µm and instead decreasing the wall thickness from an initial 800 µm down to 100 µm, in 100 µm increments every fourth pixel (Figure 6b), adjacent individual pixels begin to merge into a more continuous single image; pixels spaced further apart remain individually distinguishable. As expected, closer spacing allows multiple distinctive pixels to combine into a single macroscopic color change.

A proof of concept for more typical displays is investigated using mechanochromic HPC pixels (Figure 6c). Arrays of 500 µm^2^ square‐shaped microactuators are manufactured into a 10 by 10 “low density” (100 pixels; 1 mm spacing) and 19 by 19 “high density” (361 pixels; 250 µm spacing) configuration. The gradients in the corner of the arrays are due to the pressure supply inlet sitting close to the array and causing slight distortions. While the development of individually addressable HPC pixel arrays is beyond the scope of this work, previous reports demonstrate how this could be achieved using multiple‐layer PDMS devices.^[^ ^76^, ^77^ ^]^ For example, vertically integrated air supply inlets would enable dramatically increased pixel densities and could be driven either individually or collectively.

Lastly, by varying the HPC content (Figure 6a, top: red HPC‐water [36 wt.% H_2_O], Figure 6a, bottom: red HPC‐water [35 wt.% H_2_O]), a fine control of the observed color effect is achieved to complement the control from the applied strain and frequency, the pixel size and shape, and their arrangement as part of a larger architecture. With the addition of gels, for example, an HPC‐gelatin formulation,^[^ ^20^ ^]^ lateral flow may be suppressed at high strains, boosting the spatial resolution and thresholds described here. Furthermore, its more matte‐like appearance may potentially help if any issues such as screen glare arise. Preliminary experiments toward such devices are shown in Figure S12 (Supporting Information), however, we found that it is difficult to deposit a uniform thin layer of HPC with gel additives using our approach. Another avenue for filling the devices would be vacuum filling, where a vacuum is induced within the assembled PDMS device during injection to draw HPC from the syringe into the volume. Failing this, the adhesive properties of the PDMS film in conjunction with the fluidity of the HPC material could be considered, or indeed a new device design or actuating mechanism. This might also contribute to the sealing and mechanical robustness of HPC displays. Finally, while many mechanochromic materials are non‐toxic by virtue of being polymer‐based,^[^ ^28^ ^]^ or composites of cellulose with a mechanochromic response,^[^ ^42^ ^]^ HPC is also edible, produced at scale and is easy to formulate for mechanochromism.^[^ ^13^, ^20^ ^]^ The versatility of mechanochromic HPC is clear and is under‐researched as a potential display technology.

## 3 Conclusion

This paper shows the first investigation of aqueous HPC pixel resolutions, mechanochromic strain sensitivity, response times, and frequency responses at decreasing length scales, along with the likely practical considerations for the design of mechanochromic HPC displays. Using biodegradable, low‐cost cost and non‐toxic material as the optical component, color switching is achieved by arrays of pneumatic microactuators. We demonstrate a direct correlation between the strain applied to HPC films and the resultant color change, following a power law at strains > ≈25%. Various color changes are achieved from a single air supply, with pixels feasibly manipulated for specific effects, detail, or color through features of differing size, geometry, and architecture. We achieve switching rates of 5 Hz and rise times of ≈45–130 ms in our devices, which is insufficient to drive common display technology running at refresh rates of 60 Hz (16.7 ms per frame).^[^ ^78^, ^79^, ^80^ ^]^ However, while our devices are not optimized for achieving fast response times, we anticipate more suitable initial applications to be e‐readers, interactive displays and large scale applications such as bill boards or active wallpaper with slower response specifications.^[^ ^81^, ^82^, ^83^, ^84^ ^]^ Considerable room for improvement also exists with the careful choice of the mechanical actuators used (i.e., the actuating design principle), the HPC formulation chosen, its rheology, and optimization of the device itself.^[^ ^53^, ^85^ ^]^ These factors will determine the maximum strain, response time, frequency bandwidth, and achievable device specification. Utilizing HPC's mechanochromism may also offer insights into the complex phase and flow behavior of HPC. These devices are an initial step toward more environmentally responsible color display and pixel technologies, and we encourage the literature to investigate these concepts further.

## 4 Experimental Section

### Materials

Ultrapure Type 1 water was used for all HPC samples (Merck Millipore, Synergy System). Dry hydroxypropyl cellulose powder was supplied by NISSO Chemical Europe (HPC SSL SFP, food grade, *M_w_* 40000 g mol^−1^, D_50_: 20 µm, as reported by supplier; lot no. NHC‐4221s) at £63 per kg (excl. VAT). Trace amounts of water‐soluble nigrosine dye (Alfa Aesar, A18147) were used in all HPC formulations. PDMS was supplied by VWR International Ltd. (Sylgard 184 Elastomer Kit) at ≈£200 per kg (excl. VAT). Photomasks were supplied by JD Photodata (Film 9″ x 12″ Photomask, Grade 4). Silicon (Si) wafers were supplied by University Wafer with a <100> crystal orientation. The silanizer (1H,1H,2H,2H‐perfluorooctyltrichlorosilane 97%, CAS 78560‐45‐9) and the photodeveloper (propylene glycol methyl ether acetate (PGMEA), CAS 108‐65‐6) were provided by Sigma–Aldrich, and negative photoresist SU8‐2025 and Titanium (Ti) Primer from Microchemicals.

Rubber O‐ring chord (used as a spacer) was supplied with three cross‐sectional diameters: 1.5 mm (Simply Bearings Ltd., nitrile 70); 1 mm (Simply Bearings Ltd., nitrile 70); 0.5 mm (Polymax Ltd., nitrile 70). The glass plates (borosilicate, 10 cm^2^, 2 mm thick) were purchased from Fisher Scientific UK Ltd, the Si sealant (Everflex Aqua Mate Silicone Aquarium Sealant) from Rapid Electronics Ltd, and epoxy glue (Araldite Instant 2‐part epoxy resin) from Onecall. A biopsy punch with an internal diameter of 1.5 mm (15110‐15) was purchased from Integra Miltex, and the microfluidic tubing with an external diameter of 1.5 mm (WZ‐0 6417‐31, polytetrafluoroethylene, PTFE) from Cole‐Parmer.

Full experimental details, manufacturing, parameters, and tools could be found in Figure S1 (Supporting Information) to Figure S5 (Supporting Information) inclusive. For brevity, only key information is provided below.

### HPC Formulation

All HPC samples were formulated in a water solvent using a planetary centrifugal mixer (ThinkyMixer ARE‐250, supplied by Intertronics). The relative weights of the constituents were measured by their mass fraction (wt.%) to a total of 55 g per sample and were accurate at the time of weighing (not measured post‐formulation). All HPC samples contain 35 wt.% water unless otherwise stated, as well as 0.005 wt.% nigrosine dye to provide broadband absorption that enhances the contrast of photonic HPC's selective reflections.^[^ ^16^, ^17^, ^18^, ^21^ ^]^ Any remaining mass was comprised of sieved HPC powder using a mesh size of ≈1 mm. For efficient planetary mixing, materials were directly combined in order: water, nigrosine, and HPC, before being mixed to homogeneity using the planetary centrifugal mixer. Identical to the authors planetary centrifugal HPC formulation described elsewhere,^[^ ^20^ ^]^ three sequential mixing steps were used in a continuous manner: i) a wetting step (1600 rpm for 2 min), ii) a soak step (0 rpm of 2 min) and iii) a homogenous mixing step (1800 rpm for 2 min). After mixing, samples were poured into Falcon tubes (50 mL), whereupon they were sealed and placed into a water bath (33 °C) for 1.5 – 2.0 h to facilitate dissolution. The samples were then centrifuged (Heraeus Multifruge X1R, Thermo Scientific, 33 °C, RCF 11617 ˣg, 45 min) to remove trapped air bubbles, transferred into a syringe with luer‐lock attachment, sealed with parafilm, and submerged into a water bath (33 °C) for a further 1.5–2 h. The syringes were then stored at 4 °C for a minimum of 48 h. Before data acquisition, any air bubbles introduced during transfer into the syringe were removed via a final planetary mixing step at ≈0.35 atmospheric pressure (≈−65 kPa negative pressure gauge) in a specially designed adaptor (MTI Corporation, MSK‐PCV‐300‐LD, 1000 rpm, 2 mins clockwise, 2 mins anti‐clockwise).

No polarizers are needed to view the HPC by eye or with digital cameras. Photonic HPC is well investigated in the literature to exhibit right‐handed circularly polarized (RCP) reflection.^[^ ^20^, ^23^, ^25^, ^86^ ^]^ This is due to the right‐handed twist of its cholesteric mesophase causing a Bragg‐like circular polarisation to any reflections. If viewed through a left‐handed circularly polarized filter, the intensity is reduced significantly and has been well documented elsewhere.^[^ ^20^, ^86^ ^]^

### Photolithography

A clean Si wafer was spin‐coated with a negative photoresist (SU8‐2025), and UV‐lithography used to pattern its surface to a bespoke photomask design using the parameters detailed in (Figures S2–S5, Supporting Information). An average feature height of 52.1 µm with 2.8 µm standard deviation was achieved. A patterned Si wafer was achieved that could be used for the casting of “bulk PDMS” with embedded microchannel designs.

### PDMS Casting—Bulk PDMS

*Bulk PDMS*: The patterned Si wafer was placed in a 14 cm aluminum‐foil dish, and PDMS with a 5:1 elastomer:hardener ratio was cast on top. Air bubbles introduced from pouring were removed with gentle blowing from a plastic pipette. *PDMS Membrane*: A clean, unpatterned wafer and spin‐coater (Laurell WS‐400B‐6NPP/lite at 400 rpm for 30 s; spun twice with 1 min rest between spins) were used to manufacture 200 µm thick PDMS membranes with a 20:1 elastomer:hardener ratio. A discussion of the differential elastomer:hardener ratios is provided in (Figures S2 and S3, Supporting Information).

A pre‐heated oven (Agar, MKII Embedding Oven) then partially cures both PDMS mixtures at 65 °C (23 min for the PDMS membrane; 37 min for the bulk PDMS). Once partially cured, the bulk PDMS was cut away from its foil dish and separated from the patterned Si wafer using a sharp blade. Care must be taken not to damage the wafer to enable its reuse for future devices. Pressurized air supply inlets were then cut out of the bulk PDMS using a biopsy punch, before being rolled on to the PDMS membrane. Once joined together, the two PDMS layers were fully cured at 65 °C overnight into one cohesive device. In doing so the embedded microchannel designs of the bulk PDMS were sealed by the PDMS membrane, completing the manufacture of an inflatable microactuating membrane, or “microactuator,” and represented in Figure 1a.

### Mechanochromic Device Assembly

The fully cured PDMS device was placed (features facing up) on a Teflon sheet for ease of work (Figure S3a, Supporting Information). A rubber O‐ring chord of known thickness was placed around the outside edge of the device, acting as a spacer, with two small openings left on opposing sides for HPC injection and exit points, and a plastic luer‐lock nozzle inserted into one of the openings (Figure S3b, Supporting Information). A small volume of Si sealant was then carefully spread over the O‐ring chord and nozzle (Figure S3c, Supporting Information), and a clean glass plate pressed on top. Excess Si sealant was applied around the device to ensure a complete seal. An assembled device (without HPC) can be seen in Figure S3d (Supporting Information). The device was clamped securely with another Teflon sheet and glass plate and left to dry overnight.

Once dry the device was orientated vertically and temporarily clamped using rubber bands, lab clamps, and polystyrene grips (Figure S4a,b, Supporting Information). An HPC‐filled syringe was attached to the luer‐lock nozzle and pressurized air exerted into the syringe. Photonic HPC was injected into the device over a 15–20 min period, with the device remaining clamped to prevent any bulging of the PDMS (Figure S4c, Supporting Information). The supply pressure was increased in 20 kPa increments up to a maximum of 100 kPa until HPC comes out the opposite opening to the nozzle, whereupon both ends were sealed with epoxy glue (Figure S5, Supporting Information). Once the glue was dry, the device was ready for experiments.

### Input Signal Parameters

All experiments input signals for the pressurized air supply were applied using a square wave function, as either a single step (achieving singular HPC strain‐color response performances; Figures 1, 2, 3, 4, and 6), or as a repeating oscillating step (achieving oscillating HPC strain‐color cycling performances; Figure 5). Specific supply pressure parameters for each experiment were given where appropriate. The lowest pressure difference reliably switched by the solenoid valve is 20 kPa. The highest supply pressure was limited by breakage of the device. An upper limit of 150 kPa was set to allow reliable experiment, however, this was exceeded in Figure 6c to demonstrate the mechanochromic HPC‐PDMS devices capabilities (250 kPa). A maximum supply pressure of 300 kPa was achieved without breakage during testing.

For precision, accuracy, and repeatability of pneumatic actuation, the pressurized air supply into the PDMS microactuator was controlled electronically using custom code (Python 3). By utilizing an electronically‐controlled switch (Figure S1a, Supporting Information; solenoid switch), the microactuator was deflected and relaxed between set pressures (Figure S1a, Supporting Information; digital regulator) for a given duration or frequency. For single step measurements, a short 1 s pulse was applied while video was captured for 20 s to record the rest color baseline, actuation, and recovery of HPC back toward rest. For frequency responses involving repeated actuation, an oscillating square‐wave on/off pressure was applied at varying frequencies while the video was captured for 80 s (limited by computational processing capabilities). Long‐term cycling under controlled conditions would be needed to study the device lifetime degradation processes in HPC displays. Keeping the same input amplitude, the set frequency of the oscillation was increased sequentially (0.1, 0.5, 1, 5, or 10 Hz) and data capture repeated.

Note, at 5 Hz and above, the optical response becomes limited by the framerate of the camera (30 fps, i.e., 30 Hz) – termed aliasing – and evidenced by an oscillation appearing in the ΔHue peaks at 5 and 10 Hz (Figure 5b; Figure S10, Supporting Information). Over the course of data acquisition however, the true ΔHue values were still recorded, enabling conclusions to be drawn.

### Mechanochromic Data Acquisition

The mechanochromic response of HPC was recorded with a color USB camera (YW2307 HDMI Industrial Camera, ShenZhen YangWang Technology) and the region of interest (ROI, i.e., the pixel) processed and analyzed using FFMPEG (Version 4.4; video conversion), ImageJ (2.1.0/1.53c; ROI cropping and naming), and MATLAB_R2020a (data analysis and plotting). Between measurements, the mechanochromic device was left at rest for a minimum of 2 min to ensure the color has relaxed back to its initial rest state. Pixel RGB (red, green, blue) values from the ROI are converted into an average hue value for each frame of video using the RGB (red, green, blue) to HSL (hue, saturation, lightness) color space transformation.^[^ ^21^, ^64^, ^65^ ^]^ The first 50 frames (i.e., before any pressure was applied) within the ROI were used as the baseline hue value for the analysis of HPC's dynamic mechanochromic response (ΔHue in °). By comparing only the values of hue, any “artefacts due to different illumination conditions, which would affect only the V [i.e., L] and S parameters” were removed.^[^ ^64^, ^65^ ^]^ Fixed camera settings were also utilized to prevent any automatic color corrections by the camera, while the illumination source and camera position were fixed to eliminate any iridescent effects of HPC. All images were provided as individual frames from the captured video files.

For Figure 6b, to better observe the color change (or lack thereof) in the gaps between the pixels, a Sobel edge detection filter was applied (Image J > “Find Edges”), individually, to each image before being subtracted from its original RGB image (e.g., Figure 6b; top), producing a processed RGB image (e.g., Figure 6b; bottom). The color differences between adjacent pixels are therefore accentuated.

## Conflict of Interest

The authors declare no conflict of interest.

## Supporting information



Supporting Information

## Data Availability

The data that support the findings of this study are openly available in Apollo, Symplectic Elements, University of Cambridge at https://doi.org/10.17863/CAM.113854.
