# Supplementary material for: Mechanochromic, Low‐Cost, and Structurally Colored Displays Using Biodegradable Hydroxypropyl Cellulose
Source: Adv Mater. 2025 May 9;37(29):2418880. doi: 10.1002/adma.202418880 (PMC12288781; doi:10.1002/adma.202418880)
Supplement: Supplementary file 1 — Supporting Information [file ADMA-37-2418880-s001.pdf]

ADVANCED MATERIALS

Supporting Information

for *Adv. Mater.*, DOI 10.1002/adma.202418880

Mechanochromic, Low-Cost, and Structurally Colored Displays Using Biodegradable Hydroxypropyl Cellulose

*Charles H. Barty-King, Maxime Burgonse, Silvia Vignolini, Jeremy Baumberg and Michael De Volder**

SUPPLEMENTARY INFORMATION

Mechanochromic, low-cost, and structurally colored display using biodegradable hydroxypropyl cellulose

*Charles H. Barty-King, Maxime Burgonse, Silvia Vignolini, Jeremy Baumberg, Michael De Volder**

Department of Engineering, University of Cambridge, 17 Charles Babbage Road, Cambridge CB3 0FS (UK)

Dr. C. H. Barty-King, Dr. M. Burgonse, Prof. Jeremy Baumberg and Prof. M. De Volder
Department of Engineering, University of Cambridge, 17 Charles Babbage Road, Cambridge CB3 0FS (UK)
*email: mfld2@cam.ac.uk

Prof. S. Vignolini
Yusuf Hamied Department of Chemistry, University of Cambridge, Lensfield Road, Cambridge CB2 1EW (UK)

Keywords: hydroxypropyl cellulose, cholesteric liquid crystals, mechanochromism, reflective displays, pixels

The controlled application of strain is used to investigate the mechanochromism of HPC in a novel way. We demonstrate its value as a potential biodegradable display technology. We encourage others to develop, advance and improve on setup and results. We provide this Supplementary Information for the reader to best understand the methods and approach we use.

In this supplementary information you will find the full experimental setup and control systems (**Figure S1**), the details of the mechanochromic devices manufacture (**Figure S2** to **Figure S5** inclusive), the characterization of the PDMS membrane (microactuator) displacements along with a discussion on the strain, the common power function and its assumptions, and financial costs (**Figure S6**), color histograms and chromaticity diagrams (**Figure S7**), the shape of the ΔHue response over time (**Figure S8**), and the plots used to determine the mechanochromic relaxation time constant (**Figure S9**) for **Figure 4** of the main text. The frequency response plots that are not included in **Figure 5** of the main text are also provided (**Figure S10** and **Figure S11**). A initial attempt at injecting and analyzing HPC-gelatine formulations in the mechanochromic devices is also given (**Figure S12**).

Lastly, "**Other Design Considerations**" and opportunities for future work are discussed - feel free to use these as you like - and the Supporting Information's References given.

Further reading can also be found within the first authors PhD thesis.[1]

Figure S1 – <u>The experimental setup and control systems</u>

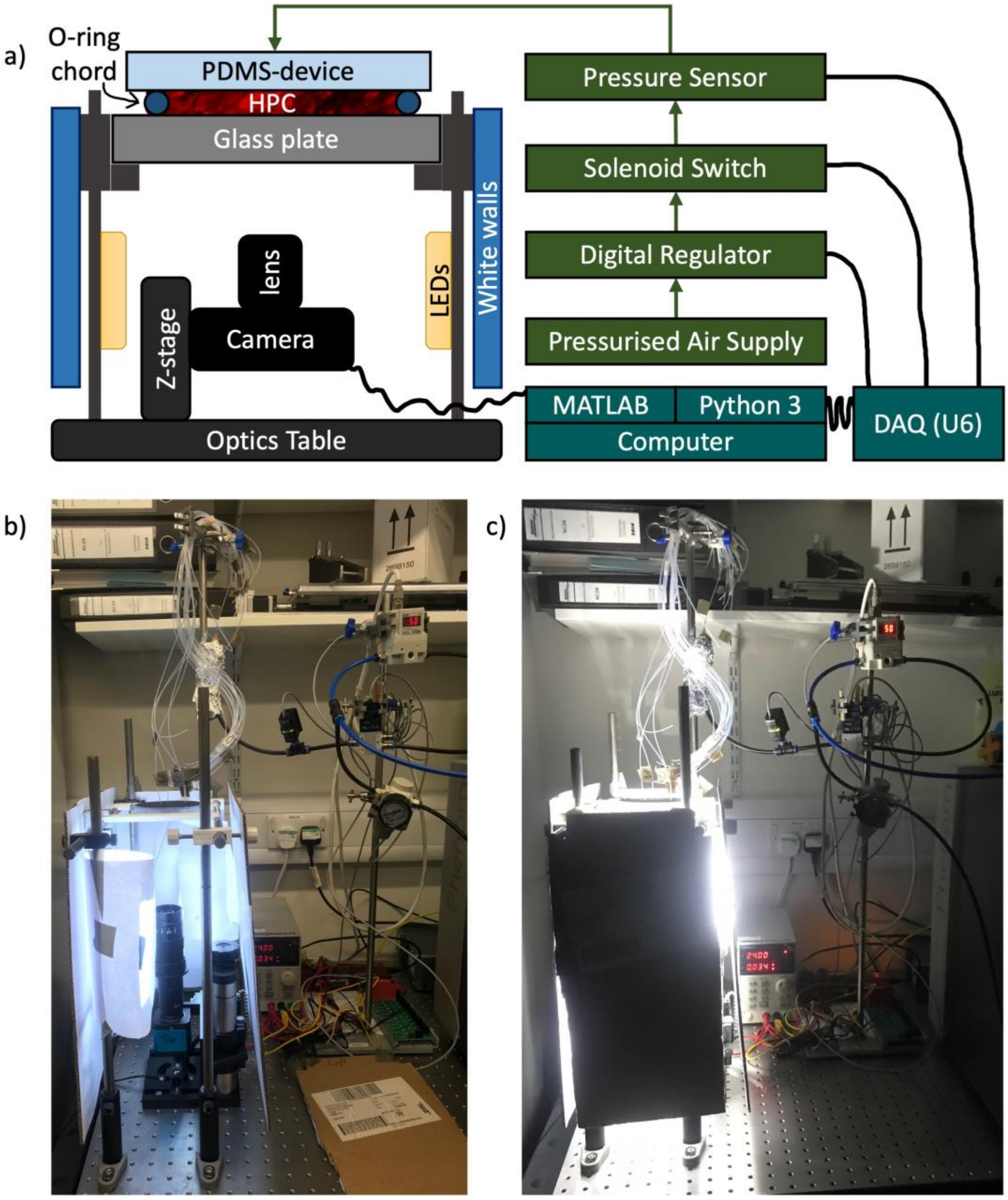

Figure S1. The experimental setup and control system for data acquisition of the mechanochromic HPC-PDMS device. **(a)** Schematic representation. Not to scale. **(b)** A picture of the experimental setup in-situ before measurement (lab lights on, rig open), and **(c)** during data acquisition (lab lights off, rig enclosed).

A USB camera (YW2307) with magnification lens attachment (180X Zoom lens C-Mount) is used to acquire 720p video footage of the mechanochromic HPC-PDMS device (**Figure S1**). Diffuse white LED lights are used as an illumination source. The air supply is controlled and recorded by a data acquisition, DAQ, system (LabJack U6, LabJack RB12 relay board, G4ODC5 output module). Maximum pressures are regulated via a digital regulator (SMC, ITV1030-21F2BS5) for accuracy over the applied pressures, and for precision control

an on-off actuation is regulated via a solenoid switch (Festo, MHE2-MS1H-3/2G-M7-K). An in-line sensor (Festo, SDE5-D10-NF-Q6E-V-K) is placed close to the main inlet of the microfluidic tubing to record the air pressure experienced by the device. The length of all tubing is kept to a minimum based on the constraints of the laboratory space. All systems are interfaced using custom code written in Python 3.

The camera is mounted on a vertical travel translation stage (Thorlabs, VAP4/M) to provide vertical movement (**Figure 1;** *z*-axis), itself mounted on an optical table, and the camera lens facing towards the ceiling. A rig is erected around the camera and all sides of the rig are covered by white walls. The top and bottom of the rig are left exposed to provide access to wires and tubing.

During experiments, the mechanochromic HPC-PDMS device is secured glass-side down in a custom 3D-printed device holder on the top of the rig, providing 2-axis lateral motion (*x* and *y*), with imaging by the camera happening beneath and looking up. A black card is placed on top of the device, with the positions of the air inlets cut out with a laser cutter, to act as an additional black contrasting background (complementing the black contrast enhancement included in the HPC formulation itself). Microfluidic tubing is then inserted into the devices air inlets from above. A bubble level is used to ensure both the camera lens and the device are positioned parallel to each other before data acquisition.

Figure S2-S5 – Manufacturing the mechanochromic HPC-PDMS device

The mechanochromic HPC-PDMS device's manufacture is schematically represented as a process flow in **Figure S2**. Materials, parameters and tools are provided in the Experimental Section of the main text, as well as the discussion below.

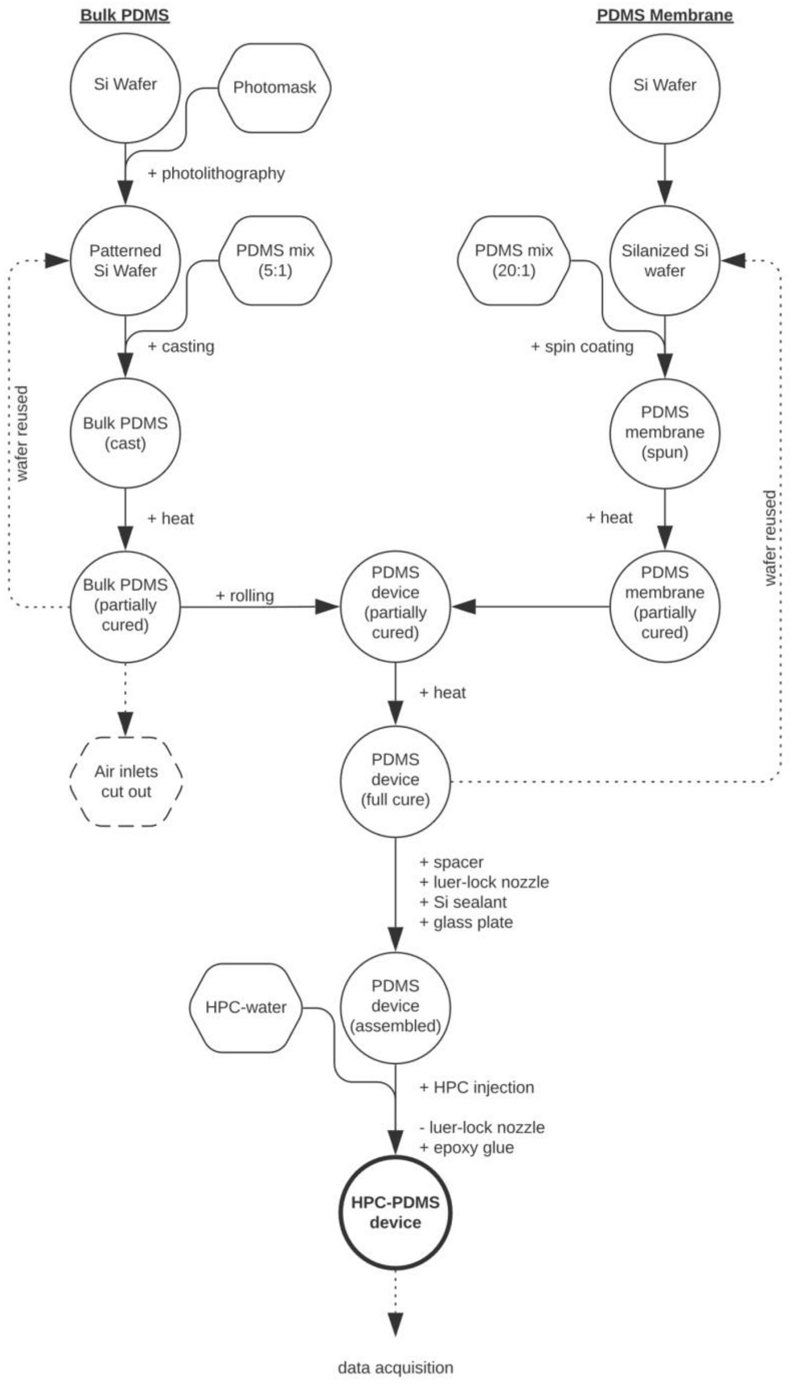

Figure S2. The schematic process flow overview describing the mechanochromic devices fabrication.

In overview, a silicon (Si) wafer is patterned to a 50 µm feature height using a photomask and UV-photolithography. A *bulk PDMS* layer is replication molded by casting

PDMS on to the patterned wafer to a thickness of 4.5 mm and partially cured. Patterned microchannels 50 μm deep are embedded into the bulk PDMS constituting the microactuator and HPC pixel designs. Once partially cured, the bulk PDMS is removed from its wafer and pressurized air supply lines ("inlets") punched out of the PDMS with a biopsy punch. A new Si wafer is silanized and spin coated with PDMS to a thickness of 200 μm and partially cured, producing a *PDMS membrane*. With great care the bulk PDMS is rolled on to the PDMS membrane that is still on its wafer, producing the *PDMS device*. The device is then fully cured to bind the two layers together and seal the microchannels, turning them into microactuators. The PDMS device is fully cured. A rubber O-ring chord of known thickness is used as a spacer around the edge of the device and sealed with a glass plate and Si sealant (**Figure S3**). HPC is injected between the PDMS device and glass plate (**Figure S4**) and sealed with epoxy glue (**Figure S5**). The complete mechanochromic *HPC-PDMS device* is driven pneumatically by switching the microactuators on and off with pressurized air,[2] supplied through inlets via microfluidic tubing and valves connected to a control system (**Figure S1**). Mechanochromism is recorded through the glass plate using a USB camera. The full manufacturing methodology is split into three: photolithography, PDMS device production and assembly, and HPC injection and sealing.

Photolithography: patterning a Si wafer

A clean room containing spin-coaters, hot plates, mask-aligners and a UV-source are used for photolithography, performed with a Karl Suss MA6 mask aligner. Baking is performed on clean hot plates, and all wafers are cleaned using water, isopropanol (IPA) and an air gun before use. A photomask provides a design template for the manufacture of the patterned Si wafer. Designs are produced using computer-aided design (CAD) software (Adobe AutoCAD 2020). A film photomask is chosen as it provides a minimum desired resolution of 20 μm (i.e., the minimum width of microchannels that connect the larger design features and pixels). A negative photoresist (SU8-2025) and its accompanying datasheet is used to obtain the spin coat, bake and exposure parameters (stated below).[3] Titanium (Ti) primer is used to increase adhesion between the photoresist SU-8 and Si wafer. A photodeveloper (propylene glycol methyl ether acetate, PGMEA) is then used to develop the patterned photoresists onto the Si wafer after UV-exposure.

First, a clean Si wafer is dehydrated at 190 °C for 5 mins and placed on a spin-coater. Excess Ti primer is poured on top and coated at 500 rpm for 10s (to spread) and 2000 rpm for 30s (to coat), covering the entire wafer surface with a uniform layer of Ti primer before being

soft baked at 95 °C for 2 mins. Excess SU8-2025 photoresist is then spread manually over the wafers surface, with particular care given to avoid the introduction of air bubbles. Any visible air bubbles are removed with the gentle blowing from a clean plastic pipette. The wafer is subsequently placed back on to the spin-coater and spun at 500 rpm for 10s (to spread) and 1750 rpm for 30s (to coat) to achieve a uniform layer of photoresist. The wafer is then soft baked at 65 °C for 3 mins and hard baked at 95 °C for 6.5 mins, ready for patterning. To pattern the wafer, it is placed into the mask aligner and exposed for 10.5 s, before undergoing a final soft bake at 65 °C for 1.5 mins and hard bake at 95 °C for 6.5 mins. To develop, the wafer is soaked in a bath of PGMEA for 330 s, washed with IPA, and rinsed with water, producing a patterned Si wafer for the subsequent casting of the bulk PDMS.

PDMS Device Production and Assembly

Bulk PDMS

The measured masses of elastomer and hardener for the bulk PDMS is calculated from the desired PDMS device thickness (4.5 mm), diameter of the casting container (14 cm foil dish), and elastomer:hardener ratio (5:1). A device thickness of 4.5 mm is chosen as it provides sufficient rigidity of the bulk PDMS, while remaining suitable flexibility to roll on to the PDMS membrane in one fluid motion (ensuring greater ease in achieving a flush connection between the two layers without any air bubbles).

First, aluminium-foil is sculpted around the outside of a 14 cm petri dish, the petri dish removed, and the patterned Si wafer placed within the foil dish. PDMS is then weighed out (5:1), mixed under vacuum in a planetary centrifugal mixer (MTI Corporation, MSK-PCV-300-LD, 1000 rpm, 2 mins clockwise, 2 mins anti-clockwise, ~ -65 kPa gauge pressure), and cast carefully into the foil dish to fully cover the patterned Si wafer. Any air bubbles introduced during casting were removed with gentle blowing from a plastic pipette. The PDMS is then placed into a pre-heated oven and partially cured (65 °C for 37 min).

Once partially cured, the bulk PDMS is cut away from the foil dish and separated from the patterned Si wafer using a sharp blade. Care is taken not to damage the wafer to enable its reuse for manufacturing more devices. A biopsy punch (Integra Miltex, 15110-15, internal diameter 1.5 mm) is used to punch inlets into the bulk PDMS, producing pressurized air supply for the microfluidic tubing (Cole Palmer, WZ-06417-31, polytetrafluoroethylene, PTFE, external diameter 1.5 mm) to be inserted into in the final device. The bulk PDMS is then ready for joining with the PDMS membrane.

PDMS membrane

The measured mass for the PDMS membrane is based on the elastomer:hardener ratio (20:1) to an excess of 15 g, before being spin-coated and poured.

First, an unpatterned Si wafer is silanized (1H,1H,2H,2H-perfluorooctyltrichlorosilane 97%, CAS 78560-45-9) in a desiccator for 1 hour, before being placed on a spin-coater (Laurell WS-400B-6NPP/lite). The reason the Si surface is silanized before spin-coating is to facilitate the eventual removal of the PDMS membrane from the wafer. Excess PDMS is weighed out to 15 g (20:1) and mixed under vacuum in a planetary centrifugal mixer (MTI Corporation, MSK-PCV-300-LD, 1000 rpm, 2 mins clockwise, 2 mins anti-clockwise, ~ 65 kPa gauge pressure). Once carefully poured onto the silanized wafer as to not introduce bubbles and spin-coated to cover the whole surface of the wafer, the PDMS membrane is partially cured (65 °C for 23 min). On removal, the PDMS membrane is ready for joining with the bulk PDMS.

PDMS elastomer:hardener ratio

The partial curing of both PDMS layers before joining provides the strongest PDMS-PDMS bond over other techniques,[4] such as oxygen plasma and corona discharge. However, the common PDMS 10:1 elastomer:hardener ratio proves ineffective at binding our bulk and membrane PDMS layers together securely.[5] Therefore, a differential 5:1 (bulk PDMS) and 20:1 (PDMS membrane) elastomer:hardener ratio is used for all devices to promote diffusion of the hardener, producing a secure binding between the two layers.

Joining the bulk PDMS and PDMS membrane together

The PDMS membrane - still on its silanized Si wafer - is laid flat on a worktop. The bulk PDMS, after being cut out of its aluminum dish, is then carefully rolled features facing down onto of the PDMS membrane in one slow-yet-fluid motion. This the most critical step of the entire manufacture and the hardest to control. Due to time and cost restraints this is done by the experienced hand of the first author. However, for high throughput manufacturing of the device a robotically controlled joining process would be worth developing.

Air bubbles trapped between the two PDMS layer during rolling were removed by using a wooden toothpick to gently guide the air bubbles away from design features (and ideally out of the device altogether). If care is not taken the design features are destroyed during this step and the device must be discarded. We recommend you take your time to roll the two layers together in a steady, fluid motion. Do not let the bulk PDMS 'come up' after contacting the PDMS membrane. This can destroy the features. We found a bulk PDMS thickness of 4.5 cm

(with an elastomer:hardener ratio of 5:1) to be ideal at providing sufficient 'bend' for careful rolling, while still maintaining macro rigidity. We also recommend you cut the bulk PDMS into a conical shape when removing it from the aluminum-dish, with the proverbial 'tip' pointing towards the patterned side of the PDMS (see **Figure S3**). This provides improved fine control when rolling by hand.

Now joined, the PDMS device is placed back into the oven and fully cured overnight at 65 °C, completing the PDMS component of the final mechanochromic device.

Device Assembly

The fully cured PDMS is removed from the silanized Si wafer and placed onto a Teflon sheet for ease of work, shown in **Figure S3a** (white sheet). Care is taken not to damage the silanized Si wafer for later reuse. Rubber O-ring chord acting as a spacer of known thickness is placed around the outside edge of the device (**Figure S3b**; 1 mm cross-sectional diameter). Two small openings are left on opposing sides to act as the HPC injection and exit points, and a plastic luer-lock nozzle placed into one of them (**Figure S3b**; green nozzle). A small volume of Si sealant is then spread across all O-ring chord (**Figure S3c**) and a clean glass plate pressed on top (**Figure S3d**). An excess of Si sealant is then applied around the outside edge of the device with a spatula to ensure a hermetic seal, including sealing in the plastic luer-lock nozzle. Another Teflon sheet is placed on top of the assembled device and secured with a weight for drying overnight. An assembled and dried PDMS device can be seen in **Figure S3d**.

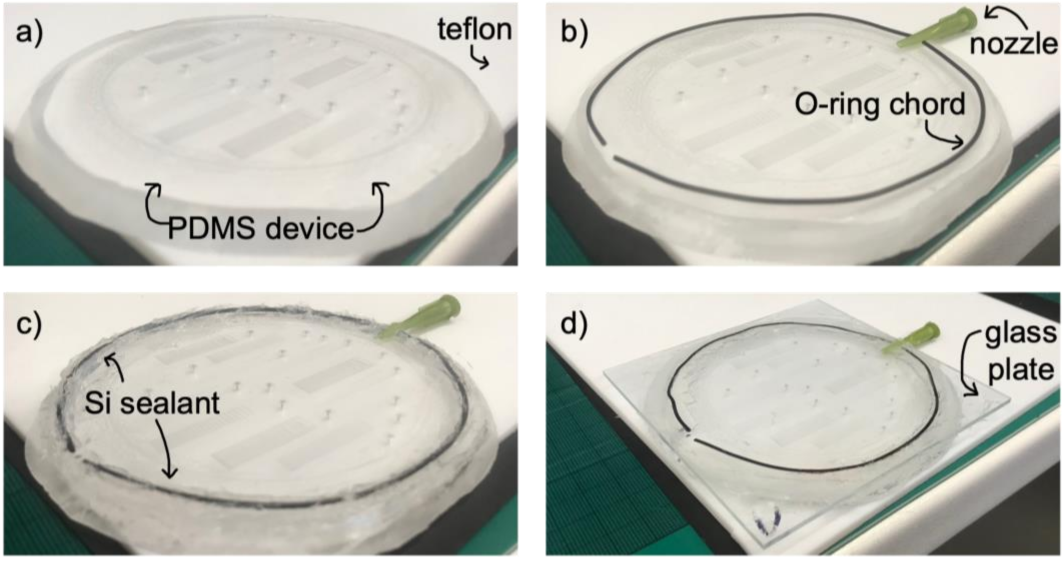

Figure S3. Images of the fully cured PDMS device. **(a)** Placed on teflon with features facing up. **(b)** Rubber O-ring chord (1 mm cross-sectional diameter) around the circumference with two openings on opposing sides and a plastic luer-lock nozzle placed into one of them. **(c)** Si sealant spread on to the O-ring chord and nozzle. **(d)** A glass plate is placed on top. The diameter of the black circle diameter is ~ 12 - 14 cm.

Injecting HPC and Sealing

With the Si sealant dry, HPC can be injected into the space between the PDMS device and glass plate (**Figure S4**). With a known cross-sectional diameter, the O-ring chord provides a known thickness to the HPC once injected. To prevent bulging of the PDMS during injection and to ensure an even spread of HPC to the desired thickness, the PDMS side of the device is clamped firmly to a second glass plate as exemplified in **Figure S4a**.

First, a Teflon sheet is laid on a spare glass plate, and the PDMS device laid on the Teflon (PDMS side down to prevent suction to the glass plate). This is then temporarily clamped using rubber bands and positioned vertically using lab clamps and polystyrene grips (**Figure S4a-b**; no additional Teflon used to enable pictures to be taken). Arbitrarily long O-ring chord with a 0.5 mm cross section could not be purchased (unlike for the 1.0 mm and 1.5 mm cross-sections). The black circles in **Figure S4** are therefore regularly manufactured O-rings with a 1 cm diameter and 0.5 mm cross-section, positioned with great care to prevent any overlap that might increase the HPC thickness. The dry Si sealant ensures any lateral gaps between the O-rings are sufficiently sealed (hermetically) on the timescales of the experiment.

A syringe containing photonic HPC is attached to the luer-lock nozzle and HPC injected into the device (**Figure S4c**). Injection parameters are described in the main text. As HPC approaches the opposite side of the device from the nozzle, the rate of injection is slowed by either reducing or removing the pressurized air supply into the syringe. Once filled, the syringe and plastic luer-lock nozzle are removed and fast drying (90 s) epoxy glue applied to both the HPC injection and exit points to seal the device.

a) b) c)

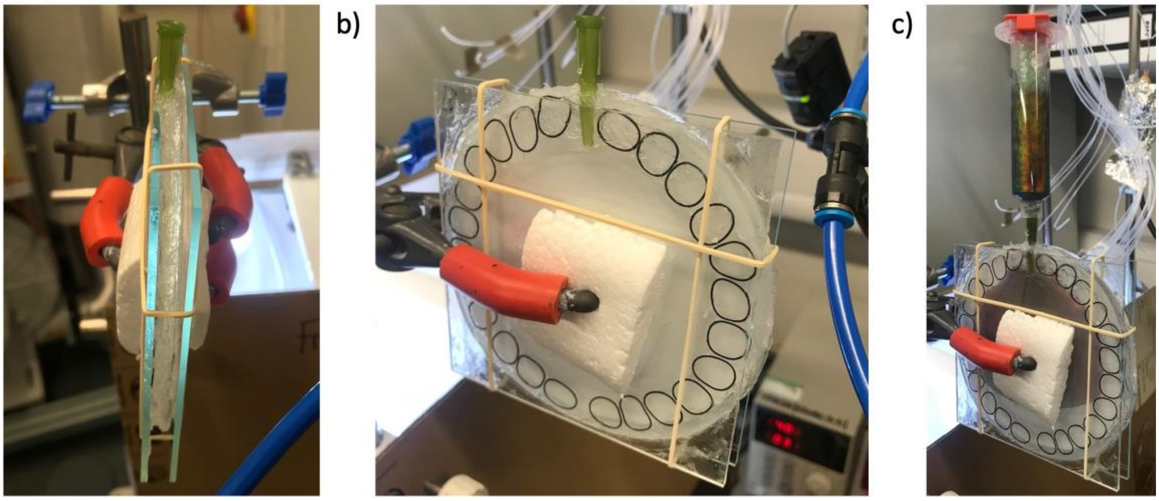

Figure S4. Images of the assembled PDMS device ready for HPC injection utilising 500 µm cross-sectional diameter O-rings (black circles) as a spacer. **(a,b)** Clamping and **(c)** HPC injection into the device.

The completed mechanochromic HPC-PDMS device (exemplified in **Figure S5**) is left for a minimum of 15 mins on a flat surface before data acquisition allow the HPC to relax to its rest coloration. The green spots shown in **Figure S5** (photographed 2 hours after data acquisition) indicate the position of the air supply inlets. The atmosphere has begun to permeate the PDMS membrane and thus dry the HPC within, causing localized blue-shifts. This could be prevented with humidity control, for example, by designing an enclosure with a water reservoir that prevents the exposure of PDMS to the atmosphere. Other solutions could include the use of hydraulic rather than pneumatic actuation of the membrane, or a device design that doesn't use pneumatics to apply mechanical strain. Within the timeframes of our experiments and with the air supply inlets located away from the pixels, this drying has no impact on our results and as such control measures are excluded. However, for longevity of an HPC display in real situations, control mechanisms and design would be needed to prevent these losses. A brief discussion of these limitations is provided at the end of the Supplementary Information.

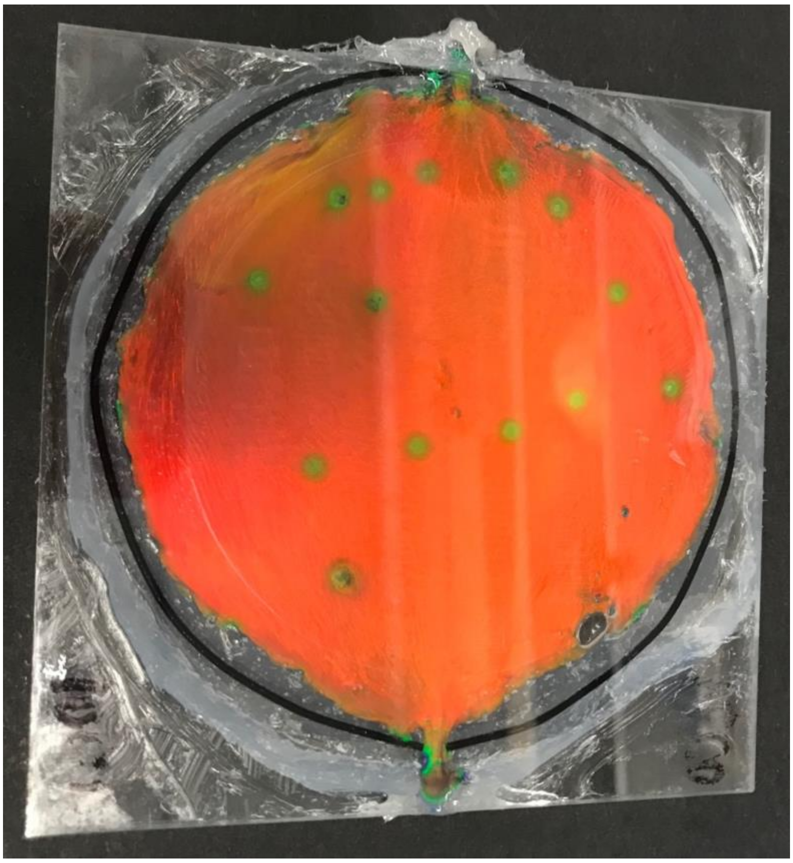

Figure S5. An image of the completed mechanochromic HPC-PDMS device 2 hours after data acquisition (4 hours after injection). Green spots are the air supply inlets where the atmosphere has begun to permeate the PDMS membrane, drying the HPC out and causing localised blue-shifts. Drying could be prevented with the use of humidity control or hydraulics.

Figure S6 and **Equation S1 –** Characterization: PDMS microactuator displacement, discussion of strain, the common power function and its assumptions, and financial costs

To quantify the relationship between the pressurized air supply and resultant strain applied to HPC, the maximum displacement of the standardized pixels PDMS membrane is measured under increasing pressure applications in air, shown in **Figure S6**.

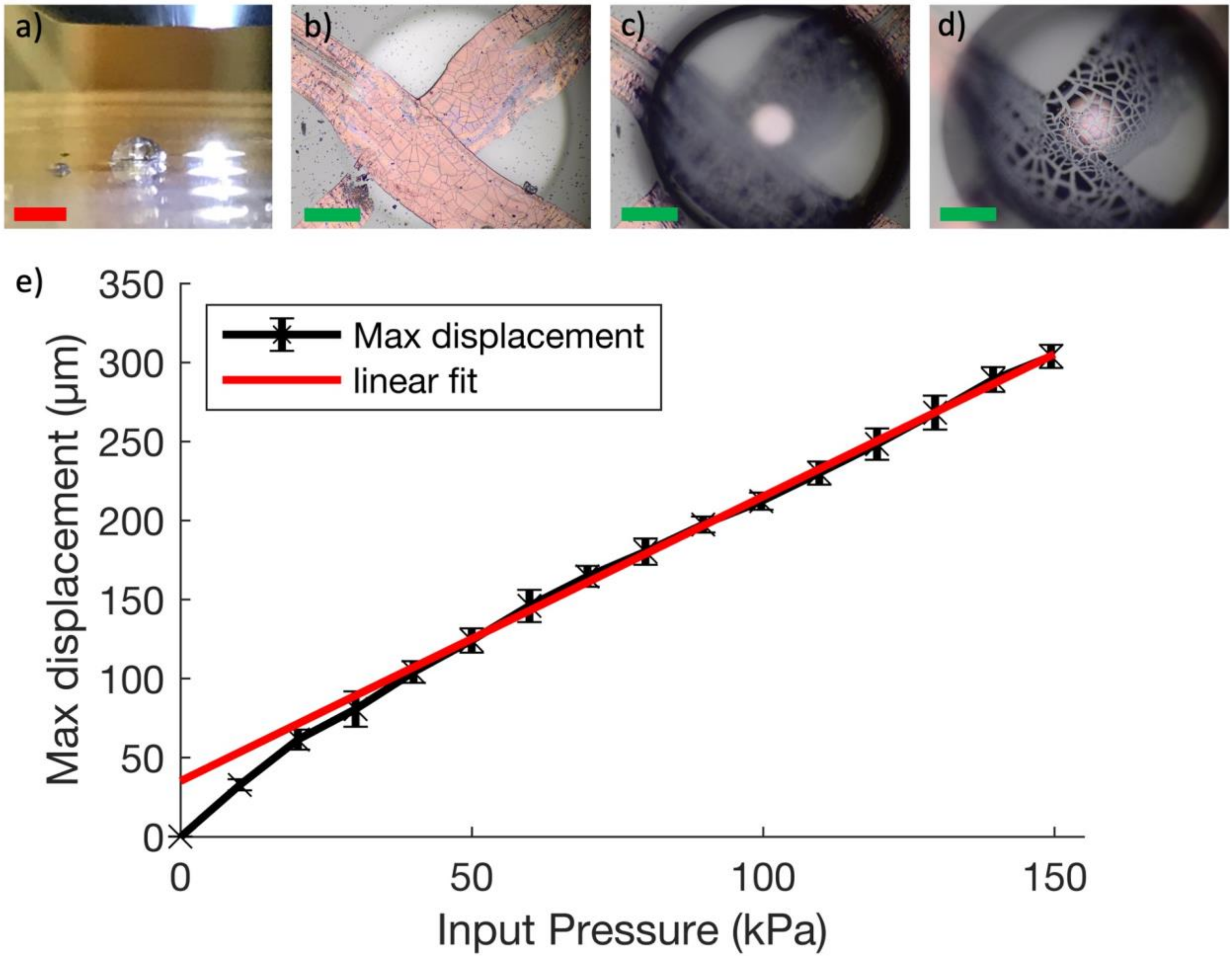

Figure S6. The standardised pixel microactuator displacements. **(a)** Macroimage. Red scale bar is 2 mm. **(b-d)** Microscope images, where: **(b)** at rest and in focus, **(c)** under actuation with the same focus as (b), **(d)** under the same actuation and re-focussed. Green scale bar is 200 μm. **(e)** A plot of the membranes maximum displacement (μm) at increasing applied pressure (kPa). Error bars are standard deviation and a linear fit above 40 kPa is applied (red line).

Using an optical microscope (Olympus BX53M) and computer-guided Z-focus stage (Marhauser Wetzlar TANGO Desktop), the microscope is focused onto deposits of black marker pen (**Figure S6b**), the membrane actuated (**Figure S6c**), and the microscope refocused (**Figure S6d**). The recorded Z-travel is then used to calculate the displacement at each applied pressure and repeated for three different devices to provide an average displacement (μm) vs input pressure (kPa) for the standardized pixel, given in **Figure S6e**. By refocusing a microscope with a motorized Z-stage onto the microactuators surface before and during a

pressure application, the maximum magnitude of the membranes displacement in air is known and used to calculate the strain as given in the main text **Equation 1**.

Figure S6e shows some non-linearity of the microactuators maximum displacement as the pressure is increased, particularly at lower pressures, and is attributed to the Young's modulus of elastomeric PDMS being non-linear with a change in its thickness,[6–8] for example, when stretched. However, the displacement is approximated to be linear above 40 kPa, in-line with the literature for deflecting constrained membranes under uniform pressure distributions.[9–11] Furthermore, to separate the mechanochromism of the features under observation from that of the air supply inlet, microchannels with different geometries (minimum width 20 µm, maximum width 100 µm, 50 µm high, various lengths) are designed into the device. No appreciable displacements (and thus mechanochromic response) are observed along the lengths of the microchannels due to their narrow 20 µm widths.

A discussion of strain, the common power function and its assumptions

By plotting the maximum ΔHue vs applied pressure for the three HPC thicknesses in **Figure 2c** of the main text, a clear linear relationship between max ΔHue vs. max ΔkPa is observed, a linear regression applied and the slope co-efficient used to quantify the mechanochromic *pressure* sensitivity of HPC in our devices. Therefore, the magnitude of HPC mechanochromism increases with decreasing HPC thickness. Being a shear-thinning liquid,[12,13] it's not obvious why HPC displays these linear properties. However, as introduced with **Equation 1** and **Figure 2**, comparative microactuator displacements (**Figure S6e**) exert a greater applied strain on thinner HPC films than thicker films. Using **Equation 1**, negative strain values are produced due to the compressive loading so a modulus term is added for plotting purposes.

To obtain the mechanochromic *strain* sensitivity of HPC as a function of its thickness, the ΔHue (°) vs. strain (%) values is plotted and shown in **Figure 2d**. Observing a power curve on the linear axes (**Figure 2d; left**), the data is plotted logarithmically (**Figure 2d; right**) to acquire the linear relationship using the common power function **Equation S1**:

$$y = a \times x^m \qquad \text{or} \qquad \log y = m \times \log x + \log a \qquad [\text{Eqn. S1}]$$

where x = strain (%), y = ΔHue (°), m = the gradient (y/x) and a = ΔHue at x = 1.[14] By taking the gradient of the logarithmic plots [Δ(log y) / Δ(log x)] in **Figure 2d (right)**, the HPC mechanochromic *strain* sensitivities of **Figure 2d (left)** are quantified. Interestingly, a good linearity exists between hue values (°) and spectral wavelengths (nm).[15]

As discussed in the main text, it is important to note that the applied strain values presented in this work are an approximation, which assume: 1) the magnitude of the PDMS membrane displacement is not influenced by the presence of HPC, and 2) HPC experiences no lateral flow during actuation. For practicalities of analysis, a further assumption is also made that the minimum thickness of HPC during compression (h_c) applies over the whole pixel. Due to difficulties in measurement, the thickness of the gap into which HPC is injected is assumed to be the cross-sectional diameters of the O-ring chords used as the spacer, providing the author with a value for HPC thickness. These assumptions combined limit the true accuracy of the final results. However, our results do give a strong indication of the relationship between color and displacement. Because of this, the tilde symbol (~) is provided for reported values throughout our paper.

As mentioned, it is assumed for the practicalities of analysis that the minimum thickness of HPC during compression (hc) applies over the whole pixel (i.e. the maximum PDMS displacement measured at any applied pressure is used to calculate the strain applied across the whole pixel). Where the bulk PDMS and PDMS membrane join at the edges of the pixel, they are in fact constrained, resulting in an arc-shaped deformation (**Figure 1b** and **Figure 2a**). A non-idealized strain and therefore non-uniform mechanochromism occurs. Edge effects are therefore observed across the pixel (**Figure 1c** and **Figure 2a**). Color histograms and chromaticity diagrams can also be found in **Figure S7** to demonstrate the color ranges we achieved.

The PDMS displacements are also measured in air only, due to difficulties in imaging in-situ. With the photonic material injected however, HPC will provide some increased resistance, making the true PDMS maximum displacement values in the completed device uncertain and likely less than reported here. Where they are used, an assumption is therefore made that they are unaffected by the presence of HPC.

We note that this approach is a very simplistic linear approximation. It is not a fully accurate measure of the forces, such as strain, acting on the mechanochromic HPC within the device. We have made these approximations due to time constraints of the work. Developing and understanding the exact relationship between the pressure applied into the device, the displacement of the PDMS membrane, and the resultant strain-induced mechanochromic response of the HPC would represent a significant value add to the work. We challenge the readers to investigate this in greater detail and with optimization. We suggest a hypothesis that mechanochromic HPC displays have the potential to compete with more established color display technology in the 60 Hz range. The data reported in this paper represents only a valuable

first initial insight into the likely practical limitations of HPC mechanochromism as an optical display and sensor technology when exploiting fine motor control at decreasing length scales.

Even with a uniform displacement across the entire pixel, aqueous HPC will still experience lateral flow of material away from the actuating PDMS during compressive strain applications.[13,16] A color-bleeding effect outside of the desired pixel will be observed. We briefly discuss lateral flow in the main text with the use of differently spaced pixels in **Figure 6**, but this represents another avenue for future work.

To those that might want to pursue an improvement on our approach, we advise the reader explore the nano-indentation literature, as well as the mechanics of inflation of flexible three-dimensional membranes.[2,10,17–21] In particular we recommend *Liu et al.* for their detailed theoretical and experimental studies of pneumatic balloon microactuators and PDMS pneumatic microactuators, respectively.[17,22] For further reading on the lateral flow of HPC, we recommend reading the work of Kamita *et al.*, Liang *et al.* and Chan *et al.*,[13,16,23] as well as the authors previous paper for how HPC-gelatine formulations may help reduce lateral flow (first results demonstrated in **Figure S12** below).[12] Further reading can also be found within the first authors PhD thesis.[1] Further potential future work is also provided below end of the Supporting Information for any reader to develop.

Financial Costs

In the main text on page 15 line 9-10 the cost to purchase HPC is stated as "£63 per kg (excl. VAT)", and the cost of PDMS "~£200 per kg (exclu. VAT)". With a maximum 10 g (being generous) of aqueous HPC material used in each device, and with HPC powder making up ~ 65 % by weight as stated in the Experimental section, the HPC in each device cost approximately £0.41p or less (i.e. £63 per kg / 1000 = £0.063p per gram; x 6.5 grams).

With a maximum 85 g of PDMS material used in each device, the PDMS per device cost approximately £8.50 p (i.e. £200 per kg / 1000 = £0.20p per ram; x 85 grams).

Our prototype therefore cost under ~ £10 per device. The reality is a bit more for laboratory and manufacturing costs, including soft lithography etc. However, the core optical component HPC is very cheap and readily available at industrial scales. The actuating mechanism used here is also arbitrary, with any sufficiently controlled microactuation of potential applicability for driving HPC display technology. The limitation to device cost will be in the choice and implementation of actuators and control systems.

Figure S7 – Color histograms and chromaticity diagrams of HPC during a 150 kPa actuation

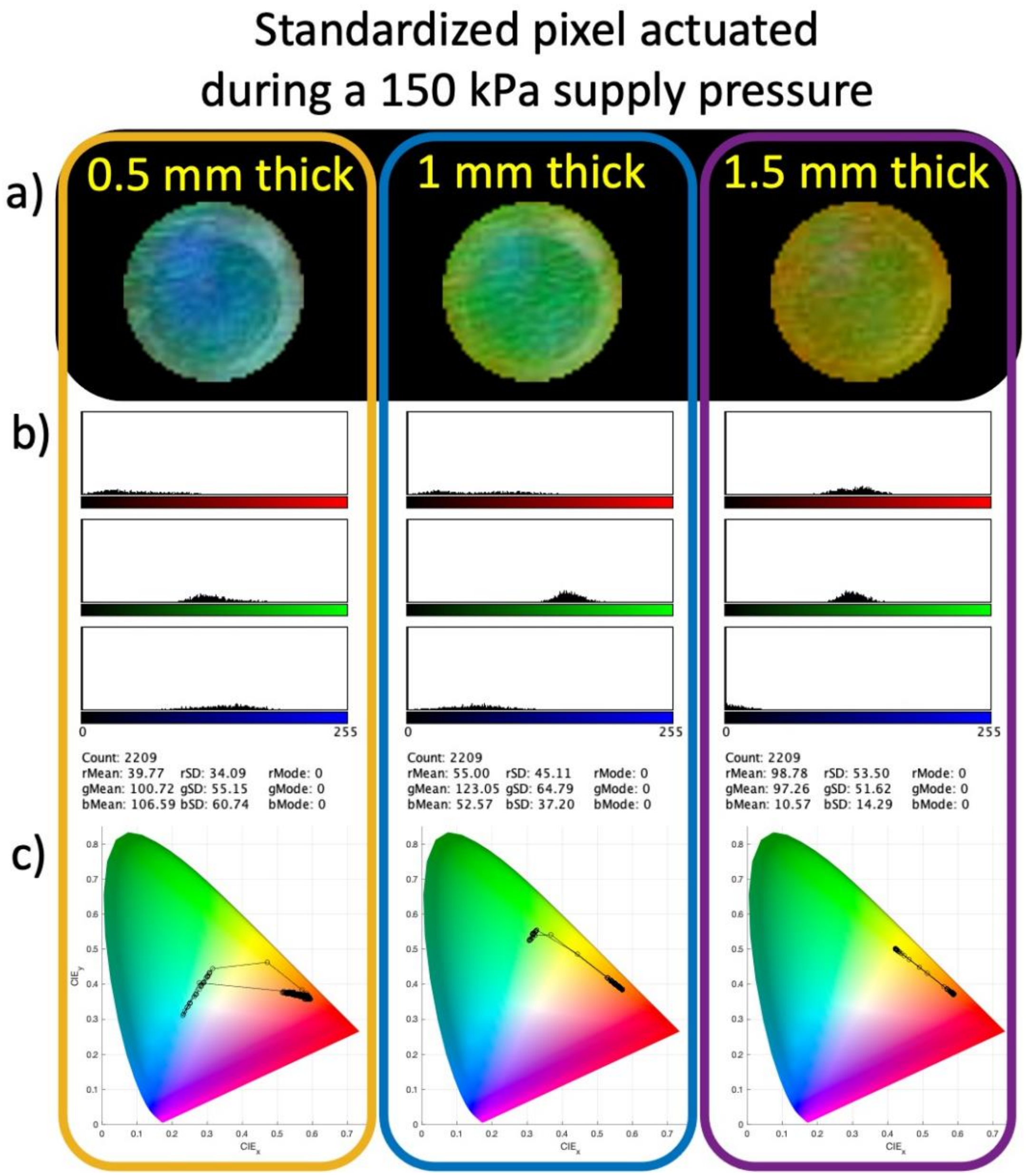

Figure S7. Mechanochromic HPC-PDMS devices at varying HPC thicknesses (yellow text) during a 150 kPa actuation. Yellow box is 0.5 mm thick HPC, blue box is 1.0 mm thick HPC, purple line is 1.5 mm thick HPC. **(a)** Pictures of the standardized pixel at the max ΔHue for each HPC thickness. Circles are 1 mm. Same as shown for **Figure 2b** of the main text. **(b)** Color histograms of (a) measured using ImageJ. **(c)** Chromaticity diagrams of (a) over the whole actuation from rest, to max ΔHue, back to rest.

The color ranges achieved using our devices and setup is shown, depicting the color ranges from the pixels given in **Figure 2** (i.e. **Figure 1c** repeated for each HPC thickness). To see the full chromaticity animations of **Figure S7c**, ".gif" files are available, along with the plotting data and code used, in the additional data from the University of Cambridge data repository (https://doi.org/10.17863/CAM.113854).

Figure S8 – Shape of ΔHue response through time

As discussed in the main text for **Figure 4**, the shape of the ΔHue response exhibits two distinct regimes through time, best exemplified in **Figure S8**. The plot on the right shows the maximum 150 kPa pulse, while the plot on the left shows the lowest 20 kPa pulse. The resulting mechanochromic response of HPC for each thickness is given in both.

The first regime is a square wave pattern that mostly follows the input signal. This is saen for the 1.5 mm thick HPC (purple line) at 150 kPa and for all thicknesses at 20 kPa. The second regime is a more instantaneous recovery after the maximum ΔHue is reached. This is seen in the 0.5 mm thick HPC (yellow line) at 150 kPa and none of the devices at 20 kPa. The 1.0 mm thick HPC (blue line) at 150 kPa demonstrates a transition between these regimes.

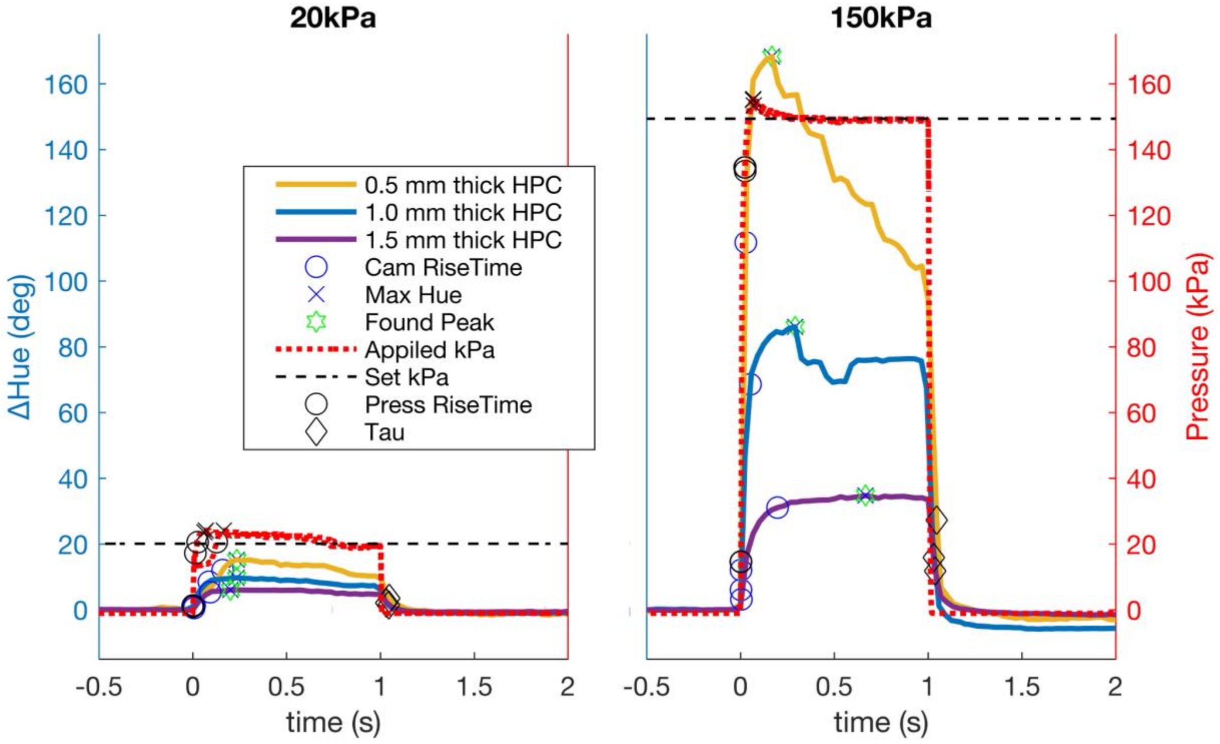

Figure S8. Time response of HPC mechanochromism given as plots of ΔHue (°) vs time (s) during independent single square pulses of 1 s (dotted red lines) at 20 kPa (left) and 150 kPa (right). Three HPC thicknesses are represented: yellow lines are 0.5 mm thick HPC, blue lines are 1.0 mm thick, and purple lines are 1.5 mm thick HPC. Circles are the rise time variables at 10% and 90% of the step height of each plot. Blue crosses are the max Hue achieved. Green Stars are the fitted peaks. Black Diamonds are the mechanochromic relaxation time constant (tau). Black Crosses are the max kPa achieved. The dashed black lines represent the supply pressure that is set before the valve to the device is opened (and the red dotted line experienced).

Figure S9 – Response time definitions and the mechanochromic relaxation time constant

All time values are normalized to the initiation of the pressure pulse, where t_0 = time zero, t_1 = the time elapsed (s) at the max pressure reached, t_2 = the time elapsed (s) at the max ΔHue reached, and t_3 = the time elapsed (s) at the end of the pressure pulse.

The **rise time** (s) is defined as the time taken for the signal to increase from 10% (o_{10}) to 90% (o_{90}) of its step height i.e., $o_{90} - o_{10}$,[24] and used as a measure of HPC's mechanochromic ability to respond to rapid, singular inputs.

The **mechanochromic relaxation time constant, τ,** (s) is defined as the time taken for the hue to fall back to 1/e (≈ 36.8 %) of its initial value,[25] normalized from the timestamp (**t_3**) of when the pressure pulse is switched off onwards, and calculated by approximating the time response of ΔHue to a first-order exponential decay.[25] This is used during the falling step due to the overshoot in ΔHue when returning to its baseline. In doing so, "the rate of recovery of the normalized Hue [from **t_3**] represents the rate of the mechanochromic relaxation back to its initially colored [**t_0**] state",[12] providing an understanding of our standardized pixels decay back to its original baseline color. Plots of ΔHue normalised from **t_3** onwards are provided in **Figure S9** (i.e. normalized from when the pressure pulse is switched off onwards), and are used to calculate the mechanochromic relaxation time constants given **Figure 4c** of the main text.

Together, these response times help to build a picture of the time responses of mechanochromic HPC in response to a single step.

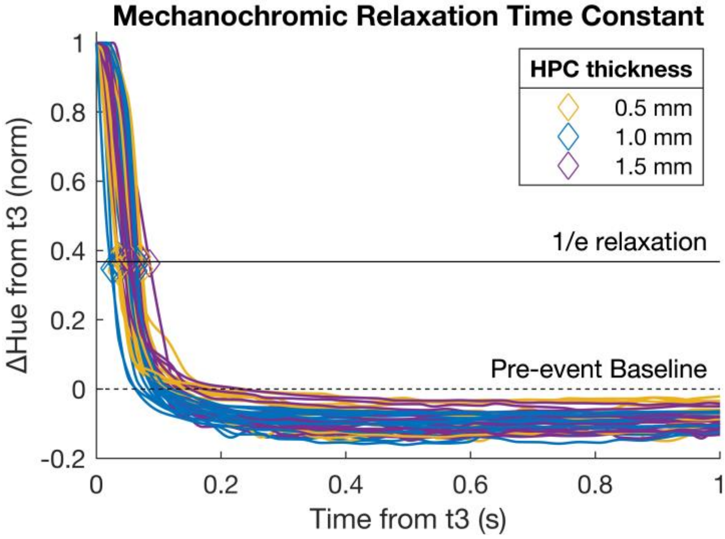

Figure S9. Mechanochromic response of HPC (normalised) vs time (s) during independent single square pulses of 1 s at increasing pressures (20-150 kPa in 10 kPa intervals) for all HPC thicknesses. The ΔHue is normalised to the last hue value before the supply pressure is removed (t3) for each plot. Black line: 1/e relaxation (i.e. ≈ 36.8 % reduction) indicating a first-order decay to the baseline colour. HPC thicknesses represented with yellow (0.5 mm), blue (1.0 mm) and purple (1.5 mm) lines.

Figure S10 – <u>All frequency responses of ΔHue as a function of time</u>

The full amplitude response of ΔHue *vs* time for each HPC thickness while a supply pressure of 100 kPa is switched on and off at frequencies of 0.1, 0.5, 1, 5 and 10 Hz. The maxima and minima of the ΔHue amplitude response are denoted with *circles* and *squares* for each oscillation respectively. The black dashed line and red dotted line denote the set and measured applied pressures, respectively. Note, at 5 Hz and above, the optical response becomes limited by the framerate of the camera (30 fps i.e., 30 Hz) - termed aliasing - and evidenced by an oscillation appearing in the ΔHue peaks at 5 and 10 Hz. Over the course of data acquisition however, the true ΔHue values are still recorded, enabling conclusions to be drawn.

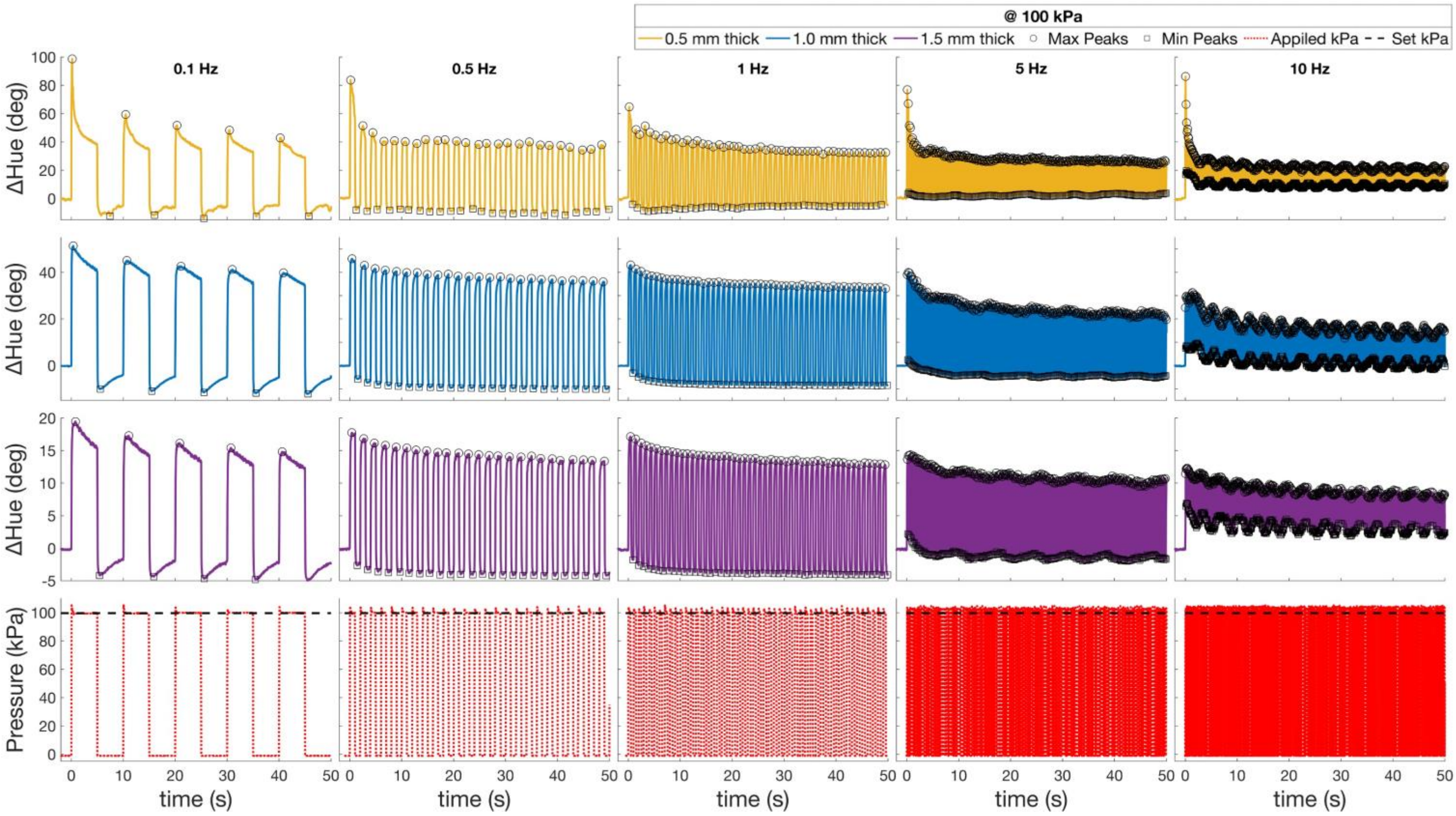

Figure S10. ΔHue (°) in response to an oscillating 100 kPa square pressure pulse (red line) switching at 0.1 Hz, 0.5 Hz, 1 Hz, 5 Hz, and 10 Hz (left to right). HPC thicknesses represented with yellow (0.5 mm), blue (1.0 mm) and purple (1.5 mm) lines. Circles and squares represent the maxima and minima peaks of the oscillating, respectively.

Figure S11 – Frequency response plots of ΔHue as a function of Hz

As discussed in the main text: "**Figure 5c** shows that under cycling conditions, as frequency increases (with HPC mechanochromism working to track the input signal and therefore becoming attenuated by HPC shear-thinning and the PDMs devices bandwidth) the maxima and minima color change begin to converge towards a common ΔHue". Demonstrated further in **Figure S11c**, at some frequency and in a sufficiently high bandwidth device, an equilibrium in the ΔHue is likely to occur that can no longer track the input signal. No further increase in frequency will alter the pixels perceived coloration, and the frequency bandwidth of HPC will have been reached. A constant HPC-pixel coloration is therefore potentially achievable.

Very stiff, high force and high frequency actuators might be able to push HPC to higher frequency bandwidths. If the true HPC bandwidth resides in or above the 50 - 60 Hz regime, photonic HPC could feasibly compete with the refresh rates of common display technology. The choice of mechanical actuation to act upon the HPC is therefore likely to be a limiting factor. To acquire the true frequency bandwidth of HPC, we would suggest providing a mechanical input signal that operates at a significantly high frequency. If the HPC mechanochromic response oscillates, its frequency bandwidth hasn't been reached. In contrast if it converges towards a constant HPC-pixel coloration then its frequency bandwidth has been reached. This might also prove a novel approach for investigating the flow behaviour of HPC through observing its mechanochromism, rather than using traditional rheological equipment.

Regardless, by optimizing HPCs rest condition as close as possible to IR, with the maximum actuated state residing in the UV, any wavelength within the visible spectrum could potentially be attained by controlling both the strain and frequency of the input signal. Further reading on the rheological properties of HPC in various formulations and solvents can be found in the literature. See the main text below **Equation 2** for numerous references.

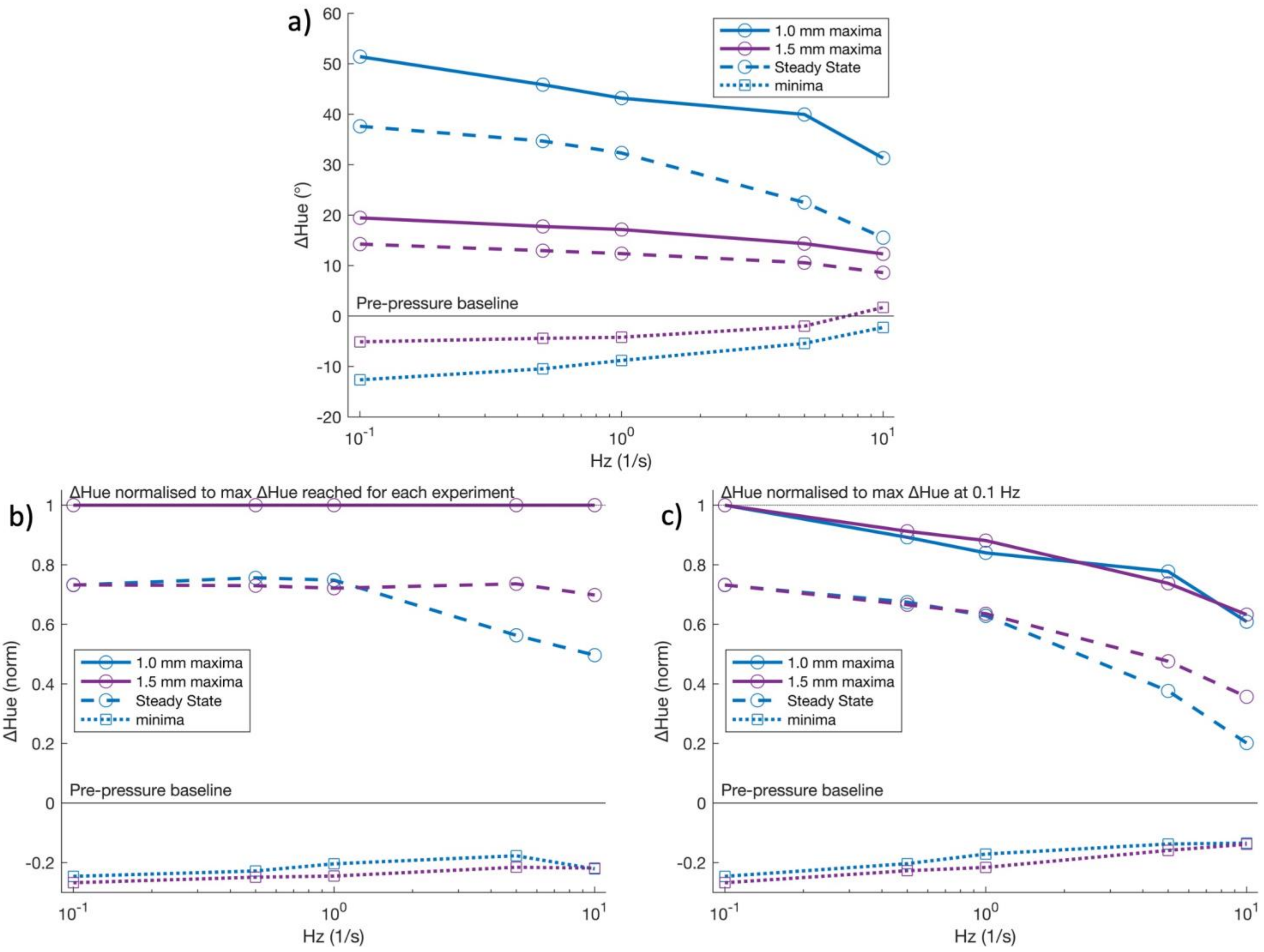

Figure S11. ΔHue vs Hz showing maxima (circles; solid line), the steady state (circles; dashed line) and minima (squares; dotted line) ΔHue for 1.5 mm thick (purple line) and 1.0 mm thick (blue line) HPC, in response to an oscillating square 100 kPa pressure pulse (red line) switching at 0.1 Hz, 0.5 Hz, 1 Hz, 5 Hz, and 10 Hz (see **Figure S10**). HPC thicknesses represented with blue (1.0 mm) and purple (1.5 mm) lines. **(a)** Absolute ΔHue (°). **(b)** ΔHue normalised to the max ΔHue reached for each frequency and HPC thickness tested. **(c)** normalised to the maximum ΔHue achieved (at 0.1 Hz) for each thickness.

Figure S12 – Attempts with formulations of HPC-gelatin-water

As the photonic HPC mesophase has a liquid behaviour,[26] by definition it cannot withstand shear forces and will flow under a mechanical stimulus. This is non-ideal for the widespread fabrication of sensors and displays. An HPC-gelatin hydrogel is therefore produced with shear-thinning behaviour that is favourable for liquid processing.[12] One mechanochromic HPC-PDMS device is manufactured with red HPC-gel [~ 35 wt% H_2O] at 1 mm thick.

Following the procedure as described for all other devices, the HPC-gel is injected into the device using a maximum 250 kPa pressure applied into the HPC-filled syringe at a frequency of 20 Hz over a 60 min period. However, the material gets clogged within the device before the HPC chamber is filled (**Figure S12a**), likely due to drying of the leading edge of the HPC (**Figure S12a:** blue-green boundary at edge of HPC). Therefore, shown in **Figure S12b-e**, only two design features are observed, the 'Pixel Size' (**Figure S12b-c**) and 'Pixel Gradient' (**Figure S12d-e**). Unfortunately, as the chamber is not filled, the HPC thickness cannot reasonably be assumed to be accurately close to the gap provided by the O-ring spacer. Furthermore, artefacts such as air bubbles and sample inhomogeneity are observed. Another avenue for filling the devices would be use vacuum injection, where a vacuum is induced within the assembled PDMS device during injection to draw HPC from the syringe into the volume. Failing this or in addition, the adhesive properties of the PDMS film in conjunction with the fluidity of the HPC material should be considered. This might also contribute to the sealing and mechanical robustness of the HPC displays.

Figure S12 shows a mechanochromic HPC-gel that has a similar optical response to its HPC-water counterpart. Suppression of the lateral flow in the HPC-gel, which dominates in HPC-water,[13,16] seems to allow for greater spatial resolution and attributed to it increased elasticity (i.e. reduced lateral flow). That's to say, the mechanochromic effects are observed below the pixel size limitation of ~ 400 – 500 μm in the main text. This finding validates the authors previous work with the HPC-gelatin system,[12] where the implications are that the mechanochromic sensitivity of HPC-gelatin is much more response to mechanical stimuli than the HPC-water system.[27] This may be helpful in optimizing the mechanochromic strain sensitivity, response times and frequency responses of HPC through an increase in the strain threshold. The dynamic range of the color range is also broadened. However, the effect in **Figure S12** may also be due to an unfilled HPC chamber or dried leading edge of the HPC (which resides close to the images shown) influencing the HPC-gels response. Lastly, its more matte-like appearance may help to prevent potential future issues such as screen glare.[12]

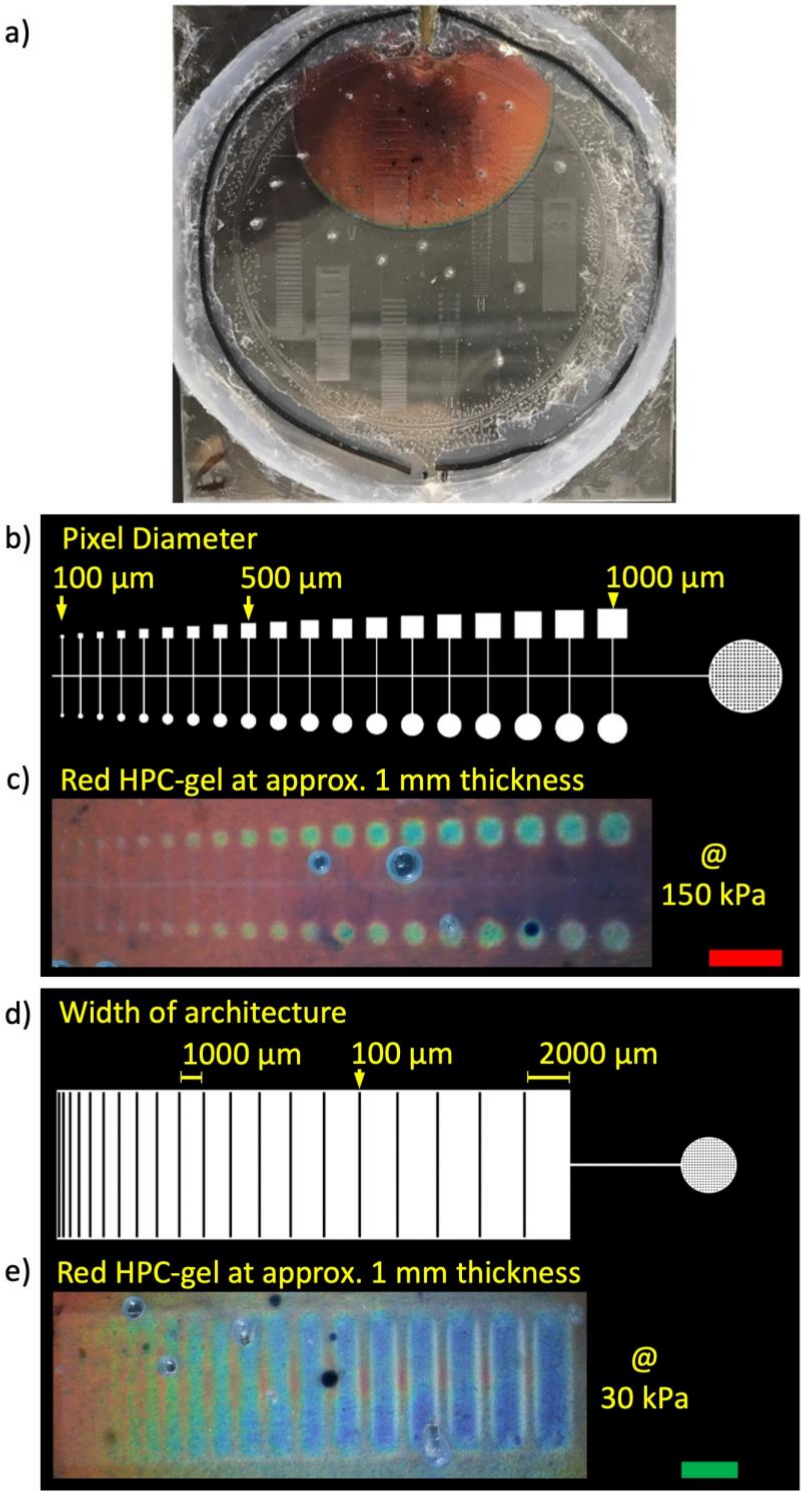

Figure S12. HPC-gel [~ 35 wt% H_2O] approximately 1 mm thick in the mechanochromic HPC-PDMS device with different design configurations. **(a)** Image after HPC injection. The blue-green line at the leading edge of the HPC indicates a drying of the material to the atmosphere, causing the incomplete filling of the HPC chamber. **(b-e)** Checkplot (white) is to scale and is the microfluidic design. Red scale bar and green scale bar are 2.5 mm. **(b)** One cohesive architecture with two pixel geometries (circles and squares) descending in diameter right-to-left from 1000 to 100 µm in 50 µm increments. **(c)** Picture at max ΔHue during 150 kPa applied pressure. **(d)** One cohesive architecture with pixels descending in width from 2000 to 100 µm in 100 µm increments with fixed spacing (100 µm). **(e)** Picture at max ΔHue during 30 kPa applied pressure.

Other Design Considerations

We suggest the most important design considerations for HPC displays is the type of actuators or actuation chosen, and the interplay between the applied strain and applied frequency. These will inform the design of any mechanochromism-driven HPC displays.

On the choice of actuators to exert mechanical forces on HPC, we chose pneumatics as our laboratory best understands nanomanufacturing techniques using PDMS. However, any actuating force that imparts mechanical strain onto HPC can be used and therefore a broad range of actuation mechanisms is possible. As in the main text, "a key challenge in achieving HPC mechanochromism while reducing pixel diameter and HPC thickness (thus shrinking device volume), is therefore likely the consistent and reliable application of strain to HPC as actuator length scales decrease". Clever design of mechanical actuation is therefore required to shrink device volume and increase pixel density. Some helpful discussion is given below.

Outside the scope of our work is the use of tensive forces to drive HPC mechanochromism (rather than the compressive forces demonstrated here). Notice that the minima ΔHue in **Figure 5**, **Figure S10** and **Figure S11** fall below the pre-strain baseline, attributed to the restorative force imparted by the relaxing microactuators. A lengthening, rather than a compression, of the HPC cholesteric pitch therefore occurs and a red-shift is observed. One could therefore optimize HPCs rest coloration to the UV and induce red-shifts into the visible spectrum towards IR regime using tensive forces. Furthermore, the faster fall in the maxima ΔHue oscillation towards a steady state, than the rise in the minima ΔHue oscillation, is likely due to the asymmetry of the reported rise and mechanochromic relaxation times in **Figure 4**, respectively. Therefore, tensive forces are likely to provide different dynamics to those reported in this paper, suggesting other routes for optimization.

Another potential innovation would be to vertically-integrate microactuators to increase the pixel density. For the early designs of our standardized pixel, the air supply inlet induced a considerable mechanochromic response into the pixels it was supposed to be investigating. This was due to the 1.5 mm diameter air supply inlet sitting too close to the pixel. To separate the mechanochromism of the pixel from that of the air supply inlet, a microchannel (20 μm wide, 50 μm high) is implemented into the final design to connect the standardized pixel to the inlet from a distance of 4.5 mm. No appreciable displacement (and thus mechanochromic response) is observed along the length of the microchannel due to its small width of 20 μm width. With vertical integration of the air inlets to supply the microactuator from beneath, the pixel density could be increased significantly as the surface area of the device isn't wasted by air inlets and supply channels.

We have also demonstrated that different mechanochromic color changes, effects and detail can be achieved using a variety of other parameters, such as initial HPC coloration, the pixel geometry and spacing, and ultimately the design of the internal architectures and features. Multiple pixels can also be controlled from a single pressure source, or be manufactured as individually-addressable pixels, providing many possible permutations that could push the boundaries of what HPC pixel display technology might achieve. Another suggestion might be to vacuum-fill HPC into a mechanochromic device to increase throughput.

Finally, there are also some considerations that may frustrate the development of HPC displays that are outside the scope of the work presented here. Firstly, mechanochromic HPC is sensitive to heat, concentration (i.e. the color changes with drying), and is iridescent (though iridescence isn't necessarily a problem for some applications and could be accounted for with testing and computation). As photonic HPC is reflective, truly integrated HPC device designs need to be lit artificially or rely on sunlight, similar to how most modern display technology requires back-lighting. However, some have successfully integrated HPC with electrical properties and controlled it's coloration in a display context.[28] The mechanochromic strain sensitivity of HPC also isn't linear; it follows a power law (**Figure 2**). An increasingly large strain magnitude is therefore required to achieve further incremental changes in hue for high strain applications. Lastly, due to lateral flow of HPC, where multiple distinctive pixels fail to combine into a single macro color change, the supply pressure could be feasibly increased to mitigate the issue where appropriate. This would induce a larger color change but also increase lateral flow outside the bounds of the pixel. Though as initial results indicate (**Figure S12**) this might be accounted for with the careful choice of HPC formulation.

Other considerations such as the use of hydraulic vs pneumatic actuation can be found in the first author's PhD thesis.[1]

Supporting Information References

[1] C. Barty-King, **2022**, DOI 10.17863/CAM.89622.
[2] M. D. Volder, D. Reynaerts, *J. Micromechanics Microengineering* **2010**, *20*, 043001.
[3] "SU-8 2000 Permanent Epoxy Resists :: MicroChem," can be found under http://www.microchem.com/Prod-SU82000.htm, **n.d.**
[4] M. A. Eddings, M. A. Johnson, B. K. Gale, *J. Micromechanics Microengineering* **2008**, *18*, 067001.
[5] T. D. C. Company, **2017**, 4.
[6] P. Bai, M. Ma, L. Sui, Y. Guo, *J. Phys. Chem. Lett.* **2021**, DOI 10.1021/acs.jpclett.1c01921.
[7] M. Liu, J. Sun, Y. Sun, C. Bock, Q. Chen, *J. Micromechanics Microengineering* **2009**, *19*, 035028.
[8] A. L. Thangawng, R. S. Ruoff, M. A. Swartz, M. R. Glucksberg, *Biomed. Microdevices* **2007**, *9*, 587.
[9] C. J. M. van Rijn, Ed., in *Membr. Sci. Technol.*, Elsevier, **2004**, pp. 111–131.
[10] S. Timoshenko, S. Woinowsky-Krieger, *Theory of Plates and Shells*, McGraw-Hill, **1959**.
[11] D. Armani, C. Liu, N. Aluru, in *Tech. Dig. IEEE Int. MEMS 99 Conf. Twelfth IEEE Int. Conf. Micro Electro Mech. Syst. Cat No99CH36291*, **1999**, pp. 222–227.
[12] C. H. Barty-King, C. L. C. Chan, R. M. Parker, M. M. Bay, R. Vadrucci, M. De Volder, S. Vignolini, *Adv. Mater.* **2021**, *33*, 2102112.
[13] H.-L. Liang, M. M. Bay, R. Vadrucci, C. H. Barty-King, J. Peng, J. J. Baumberg, M. F. L. De Volder, S. Vignolini, *Nat. Commun.* **2018**, *9*, 4632.
[14] H. Anton, I. Bivens, S. Davis, *Calculus: Early Transcendentals*, Hoboken, NJ : John Wiley & Sons, **2012**.
[15] A. Battisti, P. Minei, A. Pucci, R. Bizzarri, *Chem. Commun.* **2016**, *53*, 248.
[16] G. Kamita, B. Frka-Petesic, A. Allard, M. Dargaud, K. King, A. G. Dumanli, S. Vignolini, *Adv. Opt. Mater.* **2016**, *4*, 1950.
[17] B. Gorissen, M. D. Volder, A. D. Greef, D. Reynaerts, *Sens. Actuators Phys.* **2011**, *1*, 58.
[18] B. Gorissen, C. Van Hoof, D. Reynaerts, M. De Volder, *Microsyst. Nanoeng.* **2016**, *2*, 16045.
[19] F. De Paoli, A. Volinsky, Measuring Polydimethylsiloxane (PDMS) Mechanical Properties Using Flat Punch Nanoindentation Focusing on Obtaining Full Contact, University of South Florida, **2015**.
[20] Z. Wang, A. A. Volinsky, N. D. Gallant, *J. Appl. Polym. Sci.* **2015**, *132*, DOI 10.1002/app.41384.
[21] J. Notbohm, B. Poon, G. Ravichandran, *J. Mater. Res.* **2012**, *27*, 229.
[22] X. Liu, H. Song, W. Zuo, G. Ye, S. Jin, L. Wang, S. Li, *Energies* **2022**, *15*, 8731.
[23] C. L. C. Chan, I. M. Lei, G. T. van de Kerkhof, R. M. Parker, K. D. Richards, R. C. Evans, Y. Y. S. Huang, S. Vignolini, *Adv. Funct. Mater.* **2022**, *32*, 2108566.
[24] "Definition: rise time," can be found under https://www.its.bldrdoc.gov/fs-1037/dir-031/_4625.htm, **n.d.**
[25] B. G. Liptak, *Instrument Engineers' Handbook, Volume Two: Process Control and Optimization*, CRC Press, **2018**.
[26] R. S. Werbowyj, D. G. Gray, *Mol. Cryst. Liq. Cryst.* **1976**, *34*, 97.
[27] X. Chen, J. Chen, X. Song, T. Du, X. Deng, Z. Deng, X.-G. Hu, X. Zeng, Z. Yang, H. Yang, R. Lan, *Adv. Mater.* **n.d.**, *n/a*, 2403766.
[28] J. Wei, X. Aeby, G. Nyström, *Adv. Mater. Technol.* **2023**, *8*, 2200897.
